# Di-Silyl Rhodium(III) and Iridium(III) Complexes as Catalysts in Carbene Insertion Reactions into Hydrosilanes

**DOI:** 10.1021/acs.organomet.6c00089

**Published:** 2026-05-25

**Authors:** Adur Azpiazu-Galarza, Itxaso Bustos, Pablo Salcedo-Abraira, Claudio Mendicute-Fierro, Miguel A. Huertos

**Affiliations:** † Facultad de Química, Universidad del País Vasco (UPV/EHU), 20018 San Sebastián, Spain; ‡ Departamento de Química Inorgánica, Facultad de Ciencias, 117396 Universidad de Granada, 18071 Granada, Spain; § IKERBASQUE, Basque Foundation for Science, 48011 Bilbao, Spain

## Abstract

Catalytic carbene insertion into Si–H bonds has been recognized as an important strategy for the formation of new Si–C bonds. Herein, we report the synthesis and characterization of two new iridium­(III) complexes bearing silyl–quinoline ligands (SiN, 8-(dimethylsilyl)­quinoline): a neutral dimeric species [IrCl­(SiN)_2_]_2_ (**3**) and a cationic monomeric complex {Ir­(SiN)_2_(NCMe)_2_}­[BAr^F^_4_] (**4**). These iridium complexes, together with their rhodium analogues previously reported by our group [RhCl­(SiN)_2_] (**1**) and [Rh­(SiN)_2_(NCMe)]­[BAr^F^_4_] (**2**), were evaluated as catalysts for the insertion reaction of diazoacetates into various hydrosilanes. These studies demonstrate the catalytic activity of di-silyl rhodium­(III) and iridium­(III) complexes in carbene insertion into Si–H bonds, providing the first example of a rhodium­(III) complex catalyzing this reaction.

## Graphical abstract

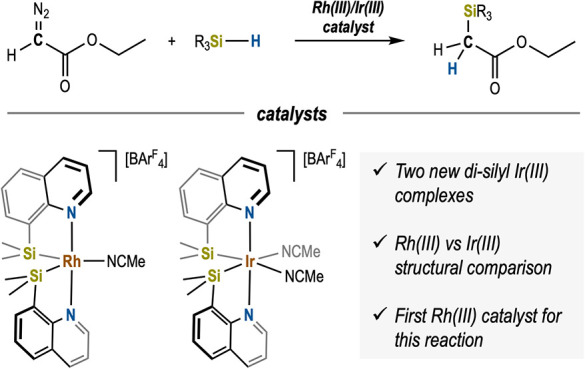

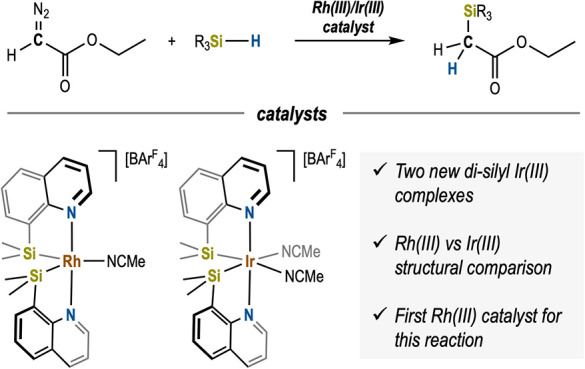

## Introduction

Transformations involving the catalytic insertion of diazo compounds into X–H (X = C, N, O, S, Si) bonds have emerged as a key strategy in organic synthesis.^1^ Because the methods used to introduce silicon motifs into organic molecules usually involve multistep synthetic routes, the insertion of diazo compounds into Si–H bonds has been postulated as an efficient approach for Si–C bond formation (Figure 1).

**Figure 1:**
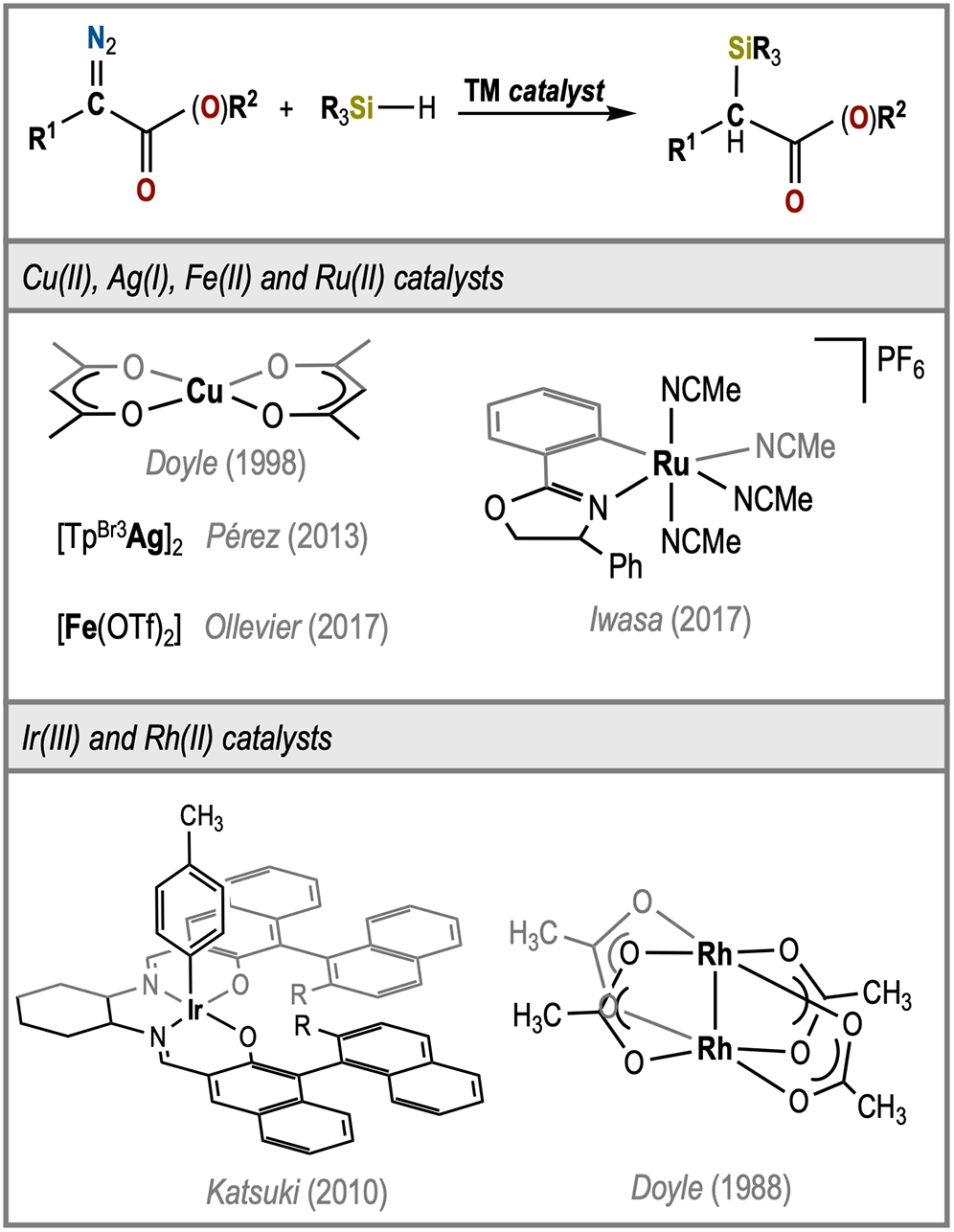
Catalytic synthesis of α-silyl ketones and α-silyl esters. Examples of transition metal (TM) complexes that catalyzed the carbene insertion into Si–H bonds.

The first example of carbene insertion into a Si–H bond was reported in 1963 by Kramer and Wright, who described the reaction of diazoalkanes with hydrosilanes under UV light or copper powder.^2^ Significant progress was made in 1988 when Doyle reported the TM-catalyzed insertion of diazo esters and diazo ketones into Si–H bonds using Rh_2_(OAc)_4_ and Cu­(acac)_2_ (Figure 1).^3^ Among these systems, Rh­(II) complexes are highly efficient, although their dinuclear nature can limit fine-tuning their reactivity. Copper catalysts have also played a prominent role in this area, with Cu­(acac)_2_, Cu­(OTf)_2_, and Cu­(I) species all showing good activity, including in asymmetric versions,^4–6^ although in some cases they show lower selectivity or a more limited substrate scope. Other TM catalytic systems have also been reported for this transformation (Figure 1). For example, Pérez and co-workers described in 2013 the use of Ag catalysts,^7^ while a Ru­(II) complex was reported by another group for the synthesis of α-silyl esters.^8^ More recently, Ollevier and co-workers showed that simple Fe­(II) salts can also efficiently promote this reaction.^9^ In addition, the use of diazo compounds also raises safety concerns, which have recently motivated the development of alternative carbene precursors.^10^ Nevertheless, diazo compounds remain highly versatile reagents, and their controlled use through improved catalytic systems continues to be of interest (Scheme 1).

More closely related to the work presented in this paper, Ir­(III) catalytic systems employing salen-type^11^ and porphyrin^12^ ligands have been developed to promote asymmetric Si–H insertion. Among all the TM used to perform this reaction, rhodium has been the most extensively studied, particularly Rh­(II) complexes such as Rh_2_(OAc)_4_ and its derivatives.^3,13^ In 2016, Xu and co-workers reported the first example of a Rh­(I) complex capable of catalyzing the insertion of diazo compounds into Si–H bonds.^14^ However, to date, no Rh­(III) complex has been reported to catalyze this reaction. In this context, Rh­(III) complexes are an unexplored oxidation state for this reaction and may offer complementary reactivity compared to Rh­(II) systems. Their mononuclear nature could also allow easier tuning of their electronic and steric properties through ligand design, leading to different catalytic behavior.

Herein, we present two cationic di-silyl-M­(III) complexes (M = Rh, Ir) capable of catalyzing the reaction between diazo esters and hydrosilanes to afford α-silyl esters.

## Results and Discussion

### Synthesis of Rhodium­(III) and Iridium­(III) Complexes

As mentioned in the introduction, this work is focused on the catalytic activity of [M­(SiN)_2_(NCMe)]­[BAr^F^_4_] {SiN, 8-(dimethylsilyl)­quinoline; M = Rh (**2**), Ir (**4**)} in the insertion of carbenes into Si–H bonds. The cationic complex **2**, synthesized from the neutral species [RhCl­(SiN)_2_] (**1**), was previously reported by the group (Figure 2a).^15^ Motivated by this result, we decided to prepare the analogous iridium complex.

**Figure 2:**
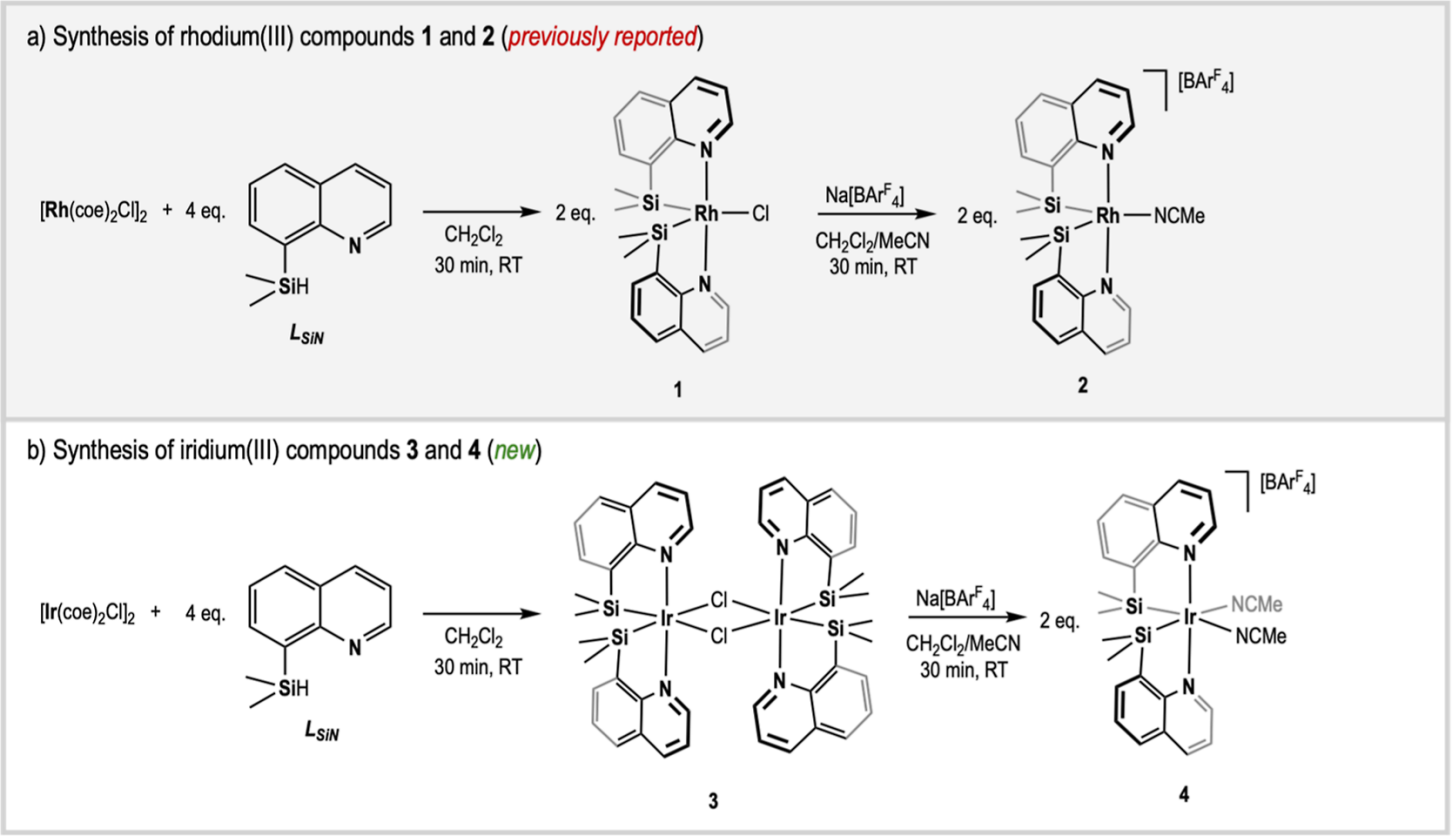
Synthesis of compounds used as catalysts in this work. (a) Synthesis of the neutral rhodium­(III) complex (**1**) and the cationic rhodium­(III) complex (**2**). These two complexes have been previously reported. (b) Synthesis of the neutral iridium­(III) complex (**3**) and the cationic iridium­(III) complex (**4**).

Reaction of [IrCl­(coe)_2_]_2_ with 4 equiv of 8-(dimethylsilane)­quinoline (**L**_**SiN**_) in CH_2_Cl_2_ at room temperature afforded, after 20 min, the neutral Ir­(III) complex [IrCl­(SiN)_2_]_2_ (**3**) in 61% yield (Figure 2b). Complex **3** was characterized in solution by NMR spectroscopy and electrospray ionization-mass spectrometry (ESI-MS). Complex **3** was also characterized in the solid state by single-crystal X-ray diffraction (Table S1, Supporting Information).

In the ^1^H NMR spectrum (Figure S.1, Supporting Information), two singlets, integral 12H, are observed for the Si–CH_3_ groups (δ 0.31 and δ −0.27). This splitting arises from the nonequivalence of the methyl groups on each silicon atom upon coordination to iridium, consistent with previous observations in similar rhodium complexes.^16^ These aforementioned signals correlate with two resonances at 3.5 and −2.4 ppm in the corresponding ^13^C­{^1^H} NMR (Figure S.2, Supporting Information). The silicon resonance at 5.0 ppm, identified through a ^1^H–^29^Si heteronuclear multiple bond correlation experiment (Figure S.15, ESI), provides evidence for Si–H oxidative addition.

Single crystals of complex **3** were obtained from a dichloromethane solution layered with pentane at room temperature. In the solid-state (Figure 3b), compound **3** features two iridium atoms bridged by two chlorine atoms, resulting in two pseudooctahedral (Tables S2 and S3, Supporting Information) sharing an edge. Two sites of the octahedral are occupied by the bridging chlorido ligands *trans* to the silicon atoms, which in turn places the nitrogen atoms in a mutual *trans* arrangement. The Ir···Ir separation of 4.035(1) Å rules out any significant metal–metal interaction. The bridging Ir–Cl bond distances [2.579(1) and 2.647(1) Å] are slightly longer than those reported for related μ-chlorido Ir­(III) complexes,^17^ consistent with the strong trans influence of the silyl ligands. The Ir–Si bond distances of 2.091(1) and 2.041(1) Å are consistent with those observed in other Ir­(III)–Si complexes.^18^ These results indicate that, in the solid state, the neutral rhodium complex **1** remains monomeric (Figure 3a), whereas the analogous iridium complex **3** adopts a dimeric structure (Figure 3b). To assess whether this metal-dependent monomer/dimer behavior is also present in solution, ^1^H diffusion-ordered spectroscopy (DOSY) NMR experiments were performed.

**Figure 3:**
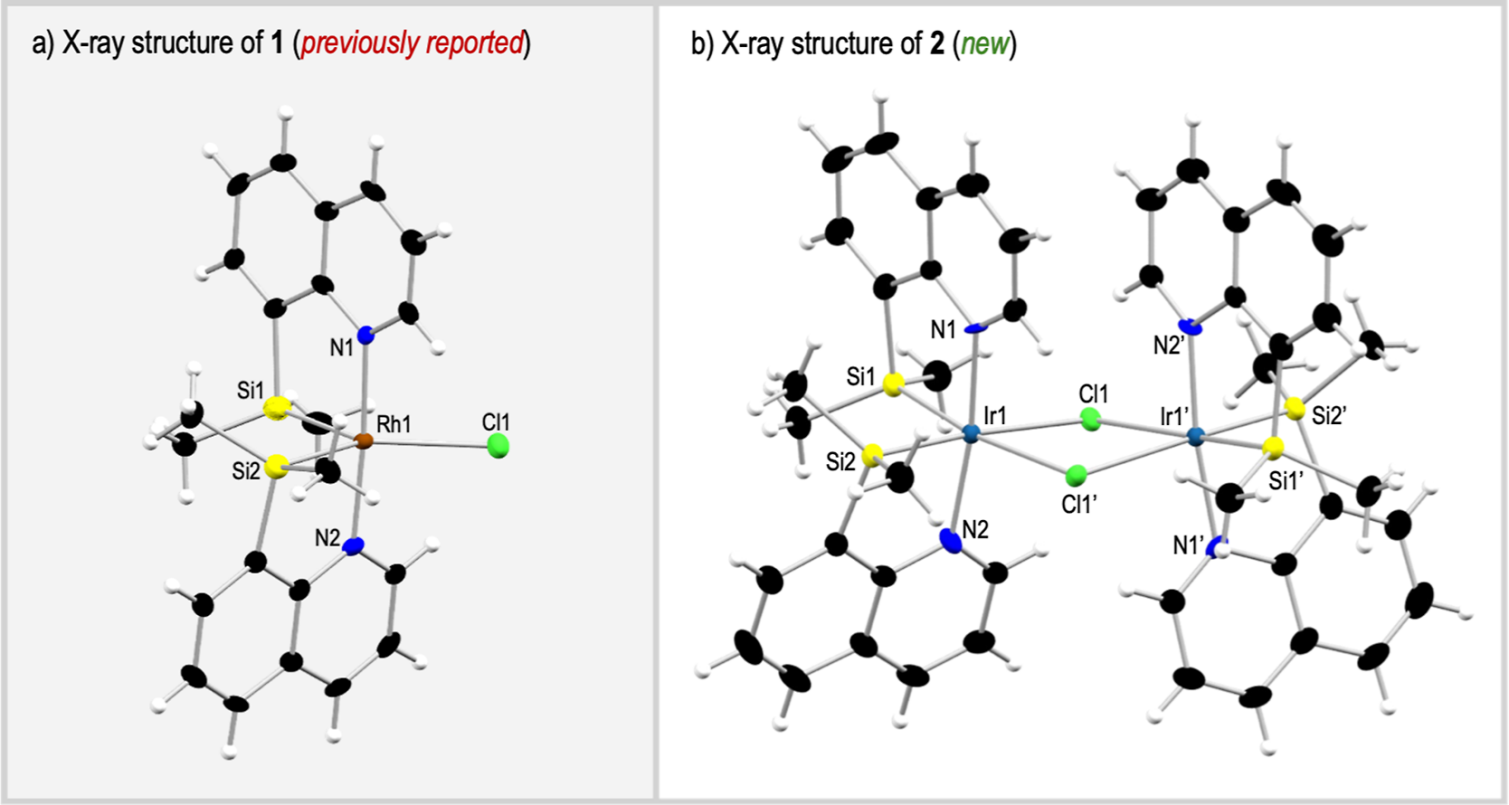
X-ray structures of complexes **1** and **3**. (a) The molecular structure of **1** has been previously reported by the same research group. (b) Molecular structure of **3**. Displacement ellipsoids are drawn at 50% probability level. Selected bond lengths [Å] and angles [°]: Ir1–N1 2.092(3), Ir1–N2 2.041(3), Ir1–Si1 2.282(1), Ir1–Si2 2.288(1), Ir1–Cl1 2.579(1), Ir1–Cl1′ 2.647(1), and N1–Ir1–N2 172.4(1).

Figure 4 shows the overlaid ^1^H DOSY spectra of the neutral complexes **1** (red) and **3** (green). As observed, complex **1** exhibits faster diffusion than complex **3**, in agreement with their monomeric and dimeric forms in solution. Additionally, the hydrodynamic radius of both molecules was calculated using the Stokes–Einstein equation, $D=\frac{k_{B}T}{6\pi \eta r_{h}}$ (where *D* = diffusion coefficient, *r*_h_ = hydrodynamic radius, *k*_B_ = Boltzmann constant, *T* = absolute temperature, and η = solvent viscosity). Using this approach, a radius of 6.12 Å was obtained for complex **1**, while complex **3** exhibited a radius of 8.38 Å. These results further support the assignment of complex **1** as monomeric and complex **3** as dimeric in solution.

**Figure 4:**
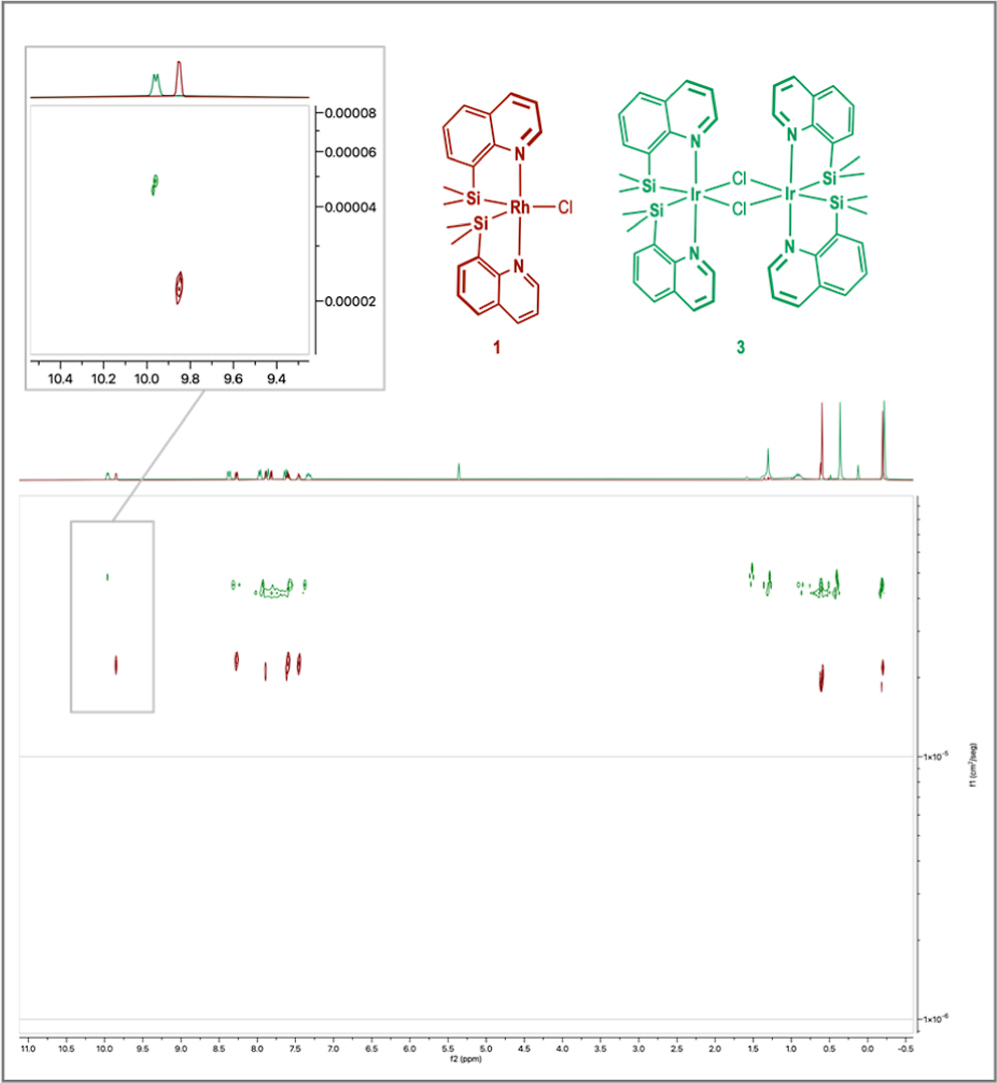
Overlaid ^1^H DOSY NMR spectra (500 MHz, CDCl_3_, 298 K) for **1** and **3**.

We decided to synthesize an iridium­(III) cationic complex similar to the previously reported [Rh­(SiN)_2_(NCMe)]­[BAr^F^_4_] (**2**),^15^ with the aim of obtaining a more efficient catalyst for carbene insertion into Si–H bonds than its neutral counterpart.

Reaction of complex **3** with two equivalents of Na­[BAr^F^_4_] in CH_2_Cl_2_ and in the presence of MeCN led to the formation of the 18-electron cationic Ir­(III) complex {Ir­(SiN)_2_(NCMe)_2_}­[BAr^F^_4_] (**4** in Figure 2). **4** was characterized in solution by NMR and ESI-MS. Unfortunately, suitable crystals were not obtained to perform a single-crystal X-ray diffraction experiment.

The ^1^H NMR spectrum (Figure S.7, Supporting Information) of **4** shows two singlet signals at 0.36 and −0.16 ppm, integrating for three protons each, which indicates the nonequivalence of the methyl groups on the silicon atoms. In the ^1^H NMR spectrum, a unique set of signals for the two quinolines and the characteristic signals of the counteranion are observed. Compound **4** exhibits a singlet signal at 2.26 ppm integrating for six protons assigned to the coordinated acetonitrile. This indicates that two acetonitrile molecules are coordinated to the metal center. NMR experiments suggest an octahedral geometry for **4**, as depicted in Figure 2. This contrasts with the rhodium analogue **2**, where only one acetonitrile ligand is coordinated, adopting a trigonal bipyramidal geometry.

This difference reflects the greater tendency of Ir­(III) to adopt octahedral geometries due to its higher ability to stabilize high coordination numbers, whereas Rh­(III) prefers 5-coordinate geometries in specific ligand arrangements.

In summary, the neutral Rh­(III) and Ir­(III) complexes **1** and **3** exhibit distinct structural features, as evidenced in the solid state by X-ray crystallography and in solution by DOSY, with the Rh complex being monomeric (hydrodynamic radius 6.12 Å) and the Ir complex tending to dimerize (hydrodynamic radius 8.38 Å). Similarly, the cationic analogues **2** and **4** show metal-dependent differences in coordination geometry and ligand environment, with the Rh complex adopting a proposed trigonal bipyramidal geometry with one coordinated MeCN and the Ir complex a proposed octahedral geometry with two coordinated MeCN, highlighting the greater tendency of Ir­(III) to stabilize higher coordination numbers.

### Catalytic Carbene Insertion into Si–H Bonds

As outlined in the Introduction, iridium­(III) complexes have been shown to be effective catalysts for carbene insertion reactions into hydrosilanes.^11,12^ In contrast, no examples have been reported for rhodium­(III) complexes catalyzing this reaction. To date, all reported rhodium-based catalysts for this transformation are rhodium­(II) and rhodium­(I) complexes.^3,13,14^

Compounds **1**–**4** were evaluated as precatalysts for the carbene insertion from ethyl diazoacetate (EDA) into triethylsilane in dichloromethane under standard reaction conditions (5 mol % of catalyst, 0.5 mmol of Et_3_SiH, and 0.1 mmol of EDA). The EDA was added dropwise to the reaction mixture over 6 h, and the reaction was allowed to proceed for an additional 10 h (total reaction time of 16 h). The conversions obtained under these conditions are listed in Table 1.

**Table 1:**
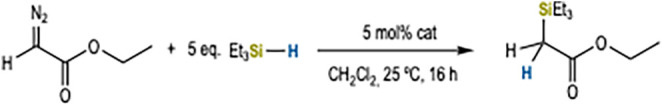
Catalytic Carbene Insertion from Ethyl Diaozoacetate into Triethylsilane^a^

entry	catalyst	conversion (%)^b^
1	**1**	-
2	**2**	58
3	**3** (2.5 mol %)^c^	-
4	**4**	98

a Reaction conditions: EDA (0.1 mmol) and triethylsilane (0.5 mmol) with 5 mol % of catalyst (0.005 mmol) in 2 mL of CH_2_Cl_2_ at room temperature.

b Conversions were calculated based on ^1^H NMR (CDCl_3_) analysis using 4-iodoanisole (0.1 mmol) as an internal standard.

c 5 mol % for the metallic center.

When neutral complexes **1** and **3** were employed, the formation of ethyl 2-(triethylsilyl)­acetate was not observed (Table 1, entries 1 and 3). The lack of reactivity of these neutral complexes may be associated with the impossibility of new ligand coordination. When the cationic iridium­(III) complex **4** was used as a catalyst, the formation of the desired product was observed with 98% conversion (Table 1, entry 4). Finally, employing the cationic rhodium­(III) complex **2** as a catalyst also led to the formation of ethyl 2-(triethylsilyl)­acetate, albeit with a more moderate conversion of 58% (Table 1, entry 2). Although the conversion obtained with complex **2** is not particularly high, this result is noteworthy as, to the best of our knowledge, it constitutes the first example of a rhodium­(III) complex catalyzing carbene insertion into Si–H bonds.

These findings motivated us to further explore iridium- and rhodium­(III)-based catalysts with other monohydrosilanes (Me_2_PhSiH, MePh_2_SiH, and Ph_3_SiH; entries 1–6 in Table 2). In all cases, as shown in entries 2, 4, and 6 of Table 2, the cationic Ir­(III) complex **4** proved to be the more efficient catalyst. Nevertheless, the Rh­(III) complex **2** is also capable of catalyzing these reactions, providing moderate to good conversions (entries 1, 3, and 5; Table 2). For both catalysts, the trend with respect to the silane employed is Me_2_PhSiH > MePh_2_SiH > Et_3_SiH > Ph_3_SiH, which suggests a balance between electronic and steric effects of the silane.

**Table 2:**
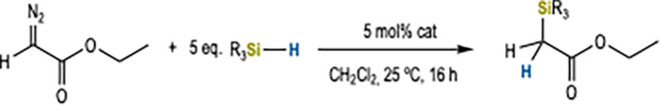
Catalytic Carbene Insertion from Ethyl Diaozoacetate into Other Silanes^a^

entry	catalyst	silane	conversion (%)^b^
1	**2**	Me_2_PhSi–H	61
2	**4**	Me_2_PhSi–H	83
3	**2**	MePh_2_Si–H	80
4	**4**	MePh_2_Si–H	95
5	**2**	Ph_3_Si–H	14
6	**4**	Ph_3_Si–H	20
7	**2**	Ph_2_HSi–H	44
8	**4**	Ph_2_HSi–H	53
9	**2**	MePhHSi–H	48
10	**4**	MePhHSi–H	57
11	**2**	PhNaphHSi–H	42
12	**4**	PhNaphHSi–H	57

a Reaction conditions: EDA (0.1 mmol) and silane (0.5 mmol) with 5 mol % of catalyst (0.005 mmol) in 2 mL of CH_2_Cl_2_ at room temperature.

b Conversions were calculated based on ^1^H NMR (CDCl_3_) analysis using 4-iodoanisole (0.1 mmol) as an internal standard.

Finally, the catalytic activity of the cationic complexes **2** and **4** was evaluated for the insertion of EDA into dihydrosilanes (Ph_2_SiH_2_, MePhSiH_2_, and NaphPhSiH_2_; entries 7–12 in Table 2). Both complexes proved to be active catalysts for this transformation, affording moderate conversions. Moreover, they were found to be selective for the insertion into only one of the two Si–H bonds present in these silanes.

The cationic Rh­(III) and Ir­(III) complexes **2** and **4** exhibit metal-dependent structural differences that correlate with their catalytic performance. Complex **2** adopts a proposed trigonal bipyramidal geometry with one coordinated MeCN, while complex **4** is proposed to be octahedral with two coordinated MeCN. The higher coordination number and octahedral geometry of **4** could create a more favorable electronic environment, potentially stabilizing carbene intermediates and enhancing substrate activation, which may lead to more efficient Si–H insertion. By contrast, the less saturated Rh­(III) complex may be more locally reactive but could offer reduced intermediate stabilization, possibly limiting its catalytic turnover. These observations suggest that variations in coordination number and geometry can influence the electronic environment of the metal and the stability of reactive intermediates, which can affect catalytic performance.

The observed activity of cationic complexes **2** and **4** in catalytic carbene insertion from EDA into different silanes prompted us to extend this study to other α-diazo esters (Table 3). Triethylsilane was selected as the model silane, and reactions were conducted under standard conditions (5 mol % catalyst, 0.5 mmol of Et_3_SiH, and 0.1 mmol of α-diazo ester). As summarized in Table 3, complexes **2** and **4** catalyze carbene insertion from methyl 2-diazopropanoate and methyl 2-diazophenylacetate into triethylsilane. Consistent with the results obtained for EDA, the cationic Ir­(III) complex **4** exhibits superior catalytic performance.

**Table 3:**
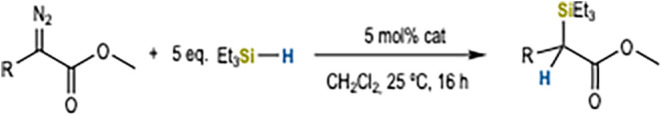
Catalytic Carbene Insertion from Methyl 2-Diazopropanoate and Methyl 2-Diazophenylacetate into Triethylsilane^a^

entry	catalyst	R	conversion (%)^b^
1	**2**	Me	7
2	**4**	Me	20
3	**2**	Ph	30
4	**4**	Ph	90

a Reaction conditions: diazoacetate (0.1 mmol) and triethylsilane (0.5 mmol) with 5 mol % catalyst (0.005 mmol) in 2 mL of CH_2_Cl_2_ at room temperature.

b Conversions were calculated based on ^1^H NMR (CDCl_3_) analysis using 4-iodoanisole (0.1 mmol) as an internal standard.

Finally, to evaluate the robustness of the catalytic system, the reaction was conducted on a 1 mmol scale by using the cationic Rh­(III) complex **2** as a representative example. Under these conditions, the corresponding product was isolated in 49% yield, confirming the efficiency of the system (Scheme 1).

**Scheme 1:**
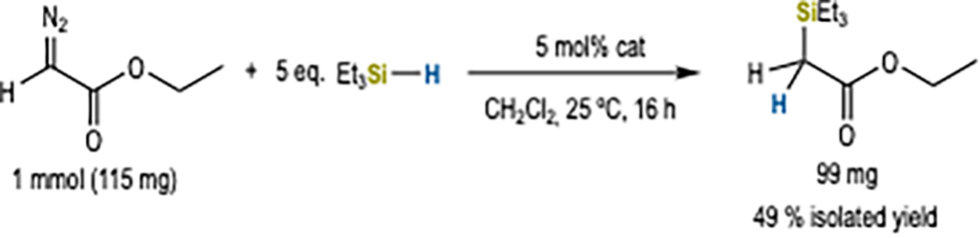
1 mmol-Scale Catalytic Carbene Insertion from EDA into Triethylsilane Catalyzed by **2**

### Proposed Mechanism for Rh­(III)-Catalyzed Carbene Insertion into Si–H Bonds

Although the mechanism for Rh­(II)-catalyzed carbene insertion into Si–H bonds is well established,^13f^ the mechanism for Rh­(III) complexes remains unknown. Nevertheless, a plausible catalytic cycle, based on a generally accepted mechanism for this class of reactions, is proposed (Figure 5). The first step of the catalytic cycle would involve coordination of the diazo substrate to the rhodium­(III) center through the negatively polarized carbon atom. This intermediate could then undergo N_2_ extrusion to generate a rhodium–carbene species. Finally, Si–H bond activation of the silane is proposed to occur via a three-center transition state involving the carbenic carbon atom. This step closes the catalytic cycle.

**Figure 5:**
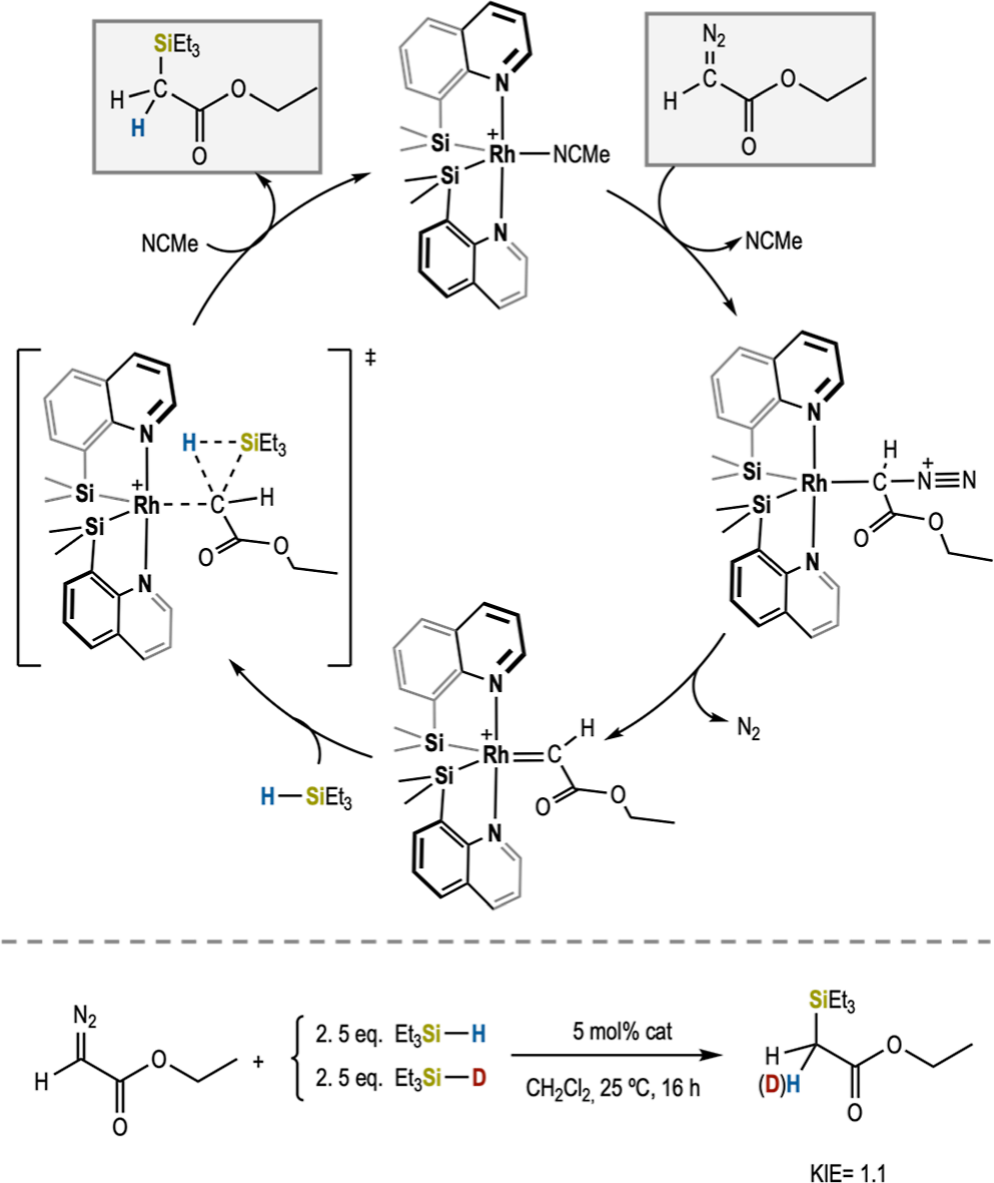
Proposed mechanism for the Rh­(III) catalyzed carbene insertion from EDA into triethylsilane (top). Kinetic isotope effect (KIE) experiment for EDA insertion into triethylsilane (bottom).

To shed light on the proposed mechanism, the KIE was investigated for the insertion reaction of EDA into triethylsilane by means of a competition experiment between Et_3_Si–H and Et_3_Si–D (Figure 5). Analysis of the crude reaction mixture by ^1^H NMR spectroscopy (Figure S.31, Supporting Information) revealed an approximate 1:0.92 ratio of ethyl 2-(triethylsilyl)­acetate/ethyl 2-(triethylsilyl)­acetate-2-*d*, corresponding to a KIE = 1.1 (Figure 5). This KIE suggest that Si–H bond activation is unlikely to be the rate-determining step of the reaction.^9,13f,19^ Additionally, a control experiment in the presence of TEMPO showed no effect on the reaction, suggesting radical pathways are unlikely.

## Conclusions

In summary, a neutral [IrCl­(SiN)_2_]_2_ (**3**) and a cationic {Ir­(SiN)_2_(NCMe)_2_}­[BAr^F^_4_] (**4**) iridium­(III) complexes were prepared and structurally characterized in solution. The neutral dimer **3** was additionally characterized in the solid state by single-crystal X-ray diffraction analysis. A structural comparison was carried out between these two new iridium­(III) complexes and their rhodium­(III) homologues, [RhCl­(SiN)_2_] (**1**) and [Rh­(SiN)_2_(NCMe)]­[BAr^F^_4_] (**2**), previously prepared by our group. This analysis shows that Ir­(III) has a stronger preference for octahedral coordination than Rh­(III). Neutral complexes **1** and **3**, as well as cationic complexes **2** and **4**, were evaluated as catalysts for carbene insertion into Si–H bonds. While neutral complexes **1** and **3** showed no catalytic activity, the cationic complexes **2** and **4** proved to be effective catalysts for this transformation. Notably, this work demonstrates the first example of a Rh­(III) catalyst able to catalyze the insertion of EDA into hydrosilanes. Based on literature data and a competitive KIE experiment, a plausible mechanism was proposed for the reaction catalyzed by cationic rhodium­(III) complex **2**.

## Supplementary Material



## Notes

Additional experimental details, NMR spectra, and X-ray single-crystal data and ESI-MS (PDF)
