# Supplementary material for: Di-Silyl Rhodium(III) and Iridium(III) Complexes as Catalysts in Carbene Insertion Reactions into Hydrosilanes
Source: Organometallics. 2026 May 25;45(11):1288–94. doi: 10.1021/acs.organomet.6c00089 (PMC13252667; doi:10.1021/acs.organomet.6c00089)
Supplement: Supplementary file 1 [file om6c00089_si_001.pdf]

Supporting Information

di-Silyl Rhodium(III) and Iridium(III) Complexes as Catalysts in Carbene Insertion Reactions into Hydrosilanes

Adur Azpiazu-Galarza,† Itxaso Bustos,† Pablo Salcedo-Abraira,§ Claudio Mendicute-Fierro† and Miguel A. Huertos*,†,‡

† Facultad de Química, Universidad del País Vasco (UPV/EHU), 20018, San Sebastián, Spain

§ Departamento de Química Inorgánica, Facultad de Ciencias, Universidad de Granada, 18071, Granada, Spain

‡ IKERBASQUE, Basque Foundation for Science, 48011, Bilbao, Spain

1. General procedures **S-2**

2. Synthesis and characterization of new Ir(III) complexes **S-3**

3. Crystallography **S-11**

4. Catalytic experiments **S-13**

5. Competition experiment for the investigation of the kinetic isotope effect (KIE) **S-23**

6. 1 mmol-scale experiment **S-24**

7. References **S-25**

1. General Procedures

All manipulations, unless otherwise stated, were performed under an argon atmosphere, using standard Schlenk techniques. Glassware was oven dried at 110ºC overnight and flamed under vacuum prior to use. Dry and oxygen free solvents were employed. $[Rh(coe)_2Cl]_2$,[1] $[Ir(coe)_2Cl]_2$,[2] $[NaBAr^F_4]$,[3] L_{SiN},[4] **1**,[5] **2**,[5] were prepared as previously described. Triethylsilane, diphenylsilane, methylphenylsilane, naphtylpheylsilane and ethyl diazoacetate were purchased from Aldrich and used without previous purification. NMR spectra were recorded on Bruker Ultra Shift 500 MHz and Bruker Advance DPX 300 MHz spectrometers. ^{1}H and ^{13}C NMR spectra were referenced to the residual solvent signals. Chemical shifts are quoted in ppm and coupling constants in Hz. Microanalysis was carried out with a LECO TRUSPEC microanalyzer. ESI-MS was recorded on LC-Q-TOF with Agilent Jet Stream ESI ionization source.

Although complexes **1** and **2** have been published by this group before, we have included their synthesis and characterization in this document to provide a more comprehensive overview.

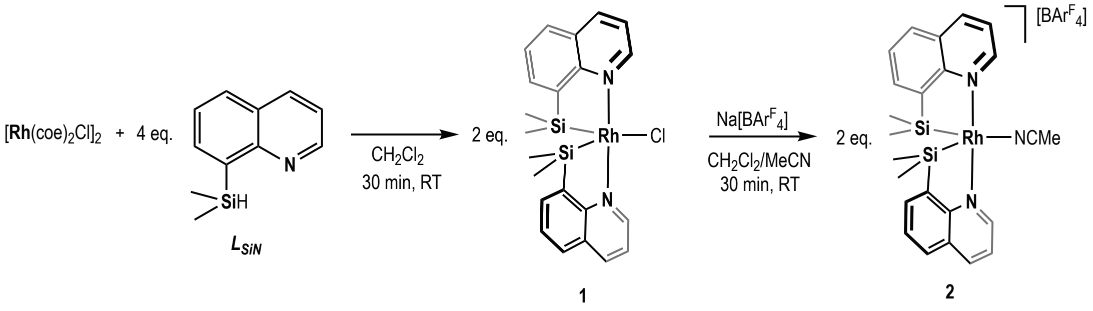

Scheme S.1. Synthesis of **1** and **2**

<u>**Characterization of 1:**</u> **^{1}H RMN (500 MHz, $CDCl_3$):** δ 9.82 (dt, $J_{1(H\text{-}H)}$ = 5.2 Hz, $J_{2(H\text{-}H)}$ = 1.4 Hz, $2H_{arom.}$), 8.24 (dd, $J_{1(H\text{-}H)}$ = 8.2 Hz, $J_{2(H\text{-}H)}$ = 1.4 Hz, $2H_{arom.}$), 7.86 (dd, $J_{1(H\text{-}H)}$ = 6.7 Hz, $J_{2(H\text{-}H)}$ = 1.4 Hz, $2H_{arom.}$), 7.79 (dd, $J_{1(H\text{-}H)}$ = 8.2 Hz, $J_{2(H\text{-}H)}$ = 1.4 Hz, $2H_{arom.}$), 7.57 (dd, $J_{1(H\text{-}H)}$ = 8.2 Hz, $J_{2(H\text{-}H)}$ = 6.7 Hz, $2H_{arom.}$), 7.42 (dd, $J_{1(H\text{-}H)}$ = 8.2 Hz, $J_{2(H\text{-}H)}$ = 5.2 Hz, $2H_{arom.}$), 0.57 (s, 6H, Si-CH_3), -0.22 (s, 6H, Si-CH_3). **$^{13}C\{^{1}H\}$ RMN (125 MHz, $CDCl_3$):** δ 158-122 ($18C_{arom.}$), 6.9 (2C, Si-CH_3), 0.4 (2C, Si-CH_3). **^{29}Si NMR (Chemical shift from ^{1}H-^{29}Si HMBC) (500 MHz, $CDCl_3$):** δ 49.2 ppm. **ESI-MS (MeCN):** calc: 475.05; found m/z 475.05. For the ion $[C_{22}H_{24}N_2RhSi_2]^+$.

<u>**Characterization of 2:**</u> **^{1}H RMN (500 MHz, CD_2Cl_2):** δ 9.12 (dt, $J_{1(H\text{-}H)}$ = 5.2 Hz, $J_{2(H\text{-}H)}$ = 1.4 Hz, $2H_{arom.}$), 8.47 (dd, $J_{1(H\text{-}H)}$ = 8.2 Hz, $J_{2(H\text{-}H)}$ = 1.4 Hz, $2H_{arom.}$), 7.98 (m, $4H_{arom.}$), 7.76 (s, $8H_{arom.}$, BAr^F_4), 7.73 (m, $2H_{arom.}$), 7.59 (s, $4H_{arom.}$, BAr^F_4) 7.55 (dd, $J_{1(H\text{-}H)}$ = 8.2 Hz, $J_{2(H\text{-}H)}$ = 5.2 Hz, $2H_{arom.}$), 2.21 (s, 3H, MeCN), 0.59 (s, 6H, Si-CH_3), -0.15 (s, 6H, Si-CH_3). **$^{13}C\{^{1}H\}$ RMN (125 MHz, CD_2Cl_2):** δ 164-117 ($42C_{arom.}$), 5.0 (2C, Si-CH_3), 2.7 (1C, MeCN), -0.7 (2C, Si-CH_3). **^{29}Si NMR (Chemical shift from ^{1}H-^{29}Si HMBC) (500 MHz, CD_2Cl_2)**: δ 52.2 ppm. **ESI-MS (MeCN):** calc: 475.05 and 516.08; found m/z 475.05 and 516.08. For the ions $[C_{22}H_{24}N_2RhSi_2]^+$ and $[C_{24}H_{27}N_3RhSi_2]^+$.

2. Synthesis and characterization of new Ir(III) complexes

Synthesis and characterization of 3

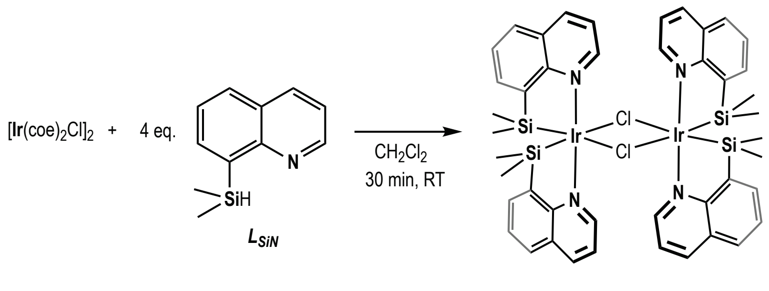

Scheme S.2. Synthesis of **3**.

To a Schlenk charged with [Ir(coe)$_2$Cl]$_2$ (100 mg, 0.12 mmol) in CH_2Cl_2 (5 ml) and 4 equivalents (90 mg, 0.48 mmol) of ligand **L1** was added. The mixture was stirred for 20 minutes and concentrated under vacuum. Addition of 20 mL of pentane gave a yellow precipitate that was washed with pentane and dried under vacuum.

<u>**Characterization of 3**</u>

Yield: 128 mg (88 %).

^{1}H RMN (500 MHz, $CDCl_3$): δ 9.92 (d, $J_{3(H-H)}$ = 5.4 Hz, 4$H_{arom.}$), 8.33 (d, $J_{3(H-H)}$ = 8.5 Hz, 4$H_{arom.}$), 7.93 (d, $J_{3(H-H)}$ = 6.9 Hz, 4$H_{arom.}$), 7.83 (dd, $J_{3(H-H)}$ = 7.3 Hz, 4$H_{arom.}$), 7.58 (dd, $J_{1(H-H)}$ = 7.8 Hz, $J_{2(H-H)}$ = 6.9 Hz, 4$H_{arom.}$), 7.29 (dd, $J_{1(H-H)}$ = 7.8 Hz, $J_{2(H-H)}$ = 5.4 Hz,4$H_{arom.}$), 0.32 (s, 12H, Si-CH_3), -0.25 (s, 12H, Si-CH_3).

^{13}C{^{1}H} RMN (125 MHz, $CDCl_3$): δ 155.1 (4$C_{arom.}$), 149.0 (4$C_{arom.}$), 136.9 (4$C_{arom.}$), 135.2 (4$C_{arom.}$), 129.1 (4$C_{arom.}$), 128.0 (4$C_{arom.}$), 127.9 (4$C_{arom.}$), 122.3 (4$C_{arom.}$), 110.3 (4$C_{arom.}$), 3.4 (4C, Si-CH_3), -2.5 (4C, Si-CH_3).

^{29}Si NMR (Chemical shift from ^{1}H-^{29}Si HMBC) (500 MHz, $CDCl_3$): δ 5.2 ppm.

ESI-MS (MeCN): calc: 565.11; found m/z 565.11. For the ion $[C_{22}H_{24}N_2IrSi_2]^+$.

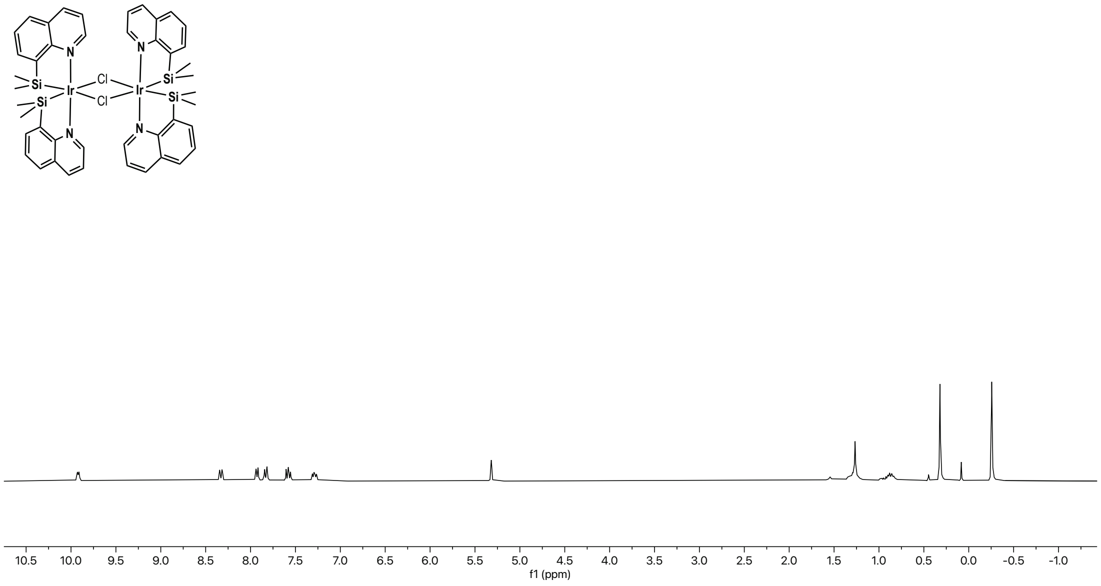

Figure S.1. ^{1}H NMR of **3.**

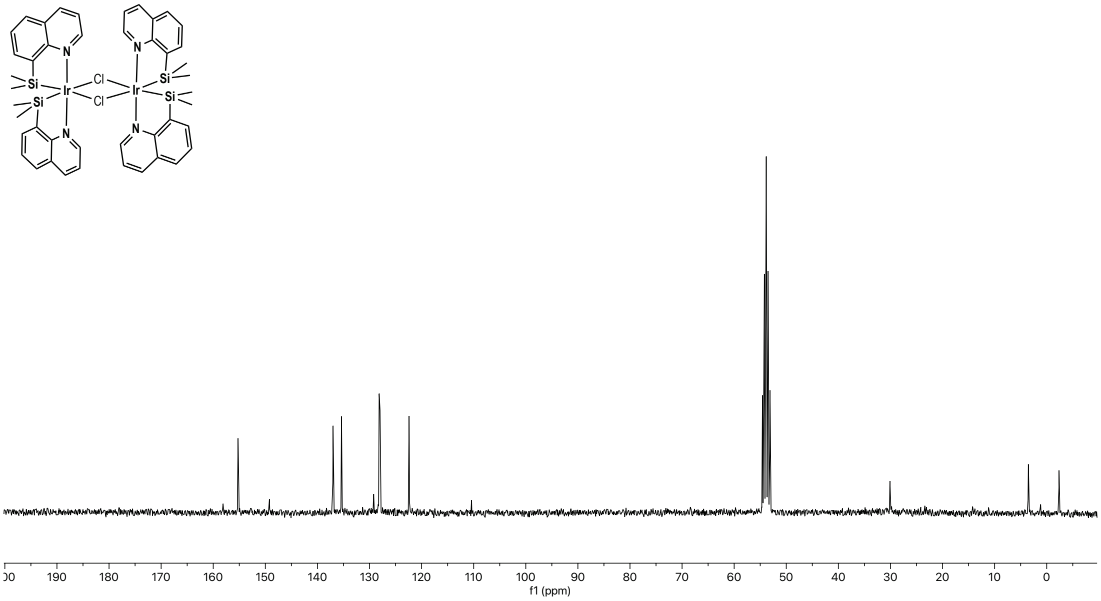

Figure S.2. $^{13}C\{^{1}H\}$ NMR of **3.**

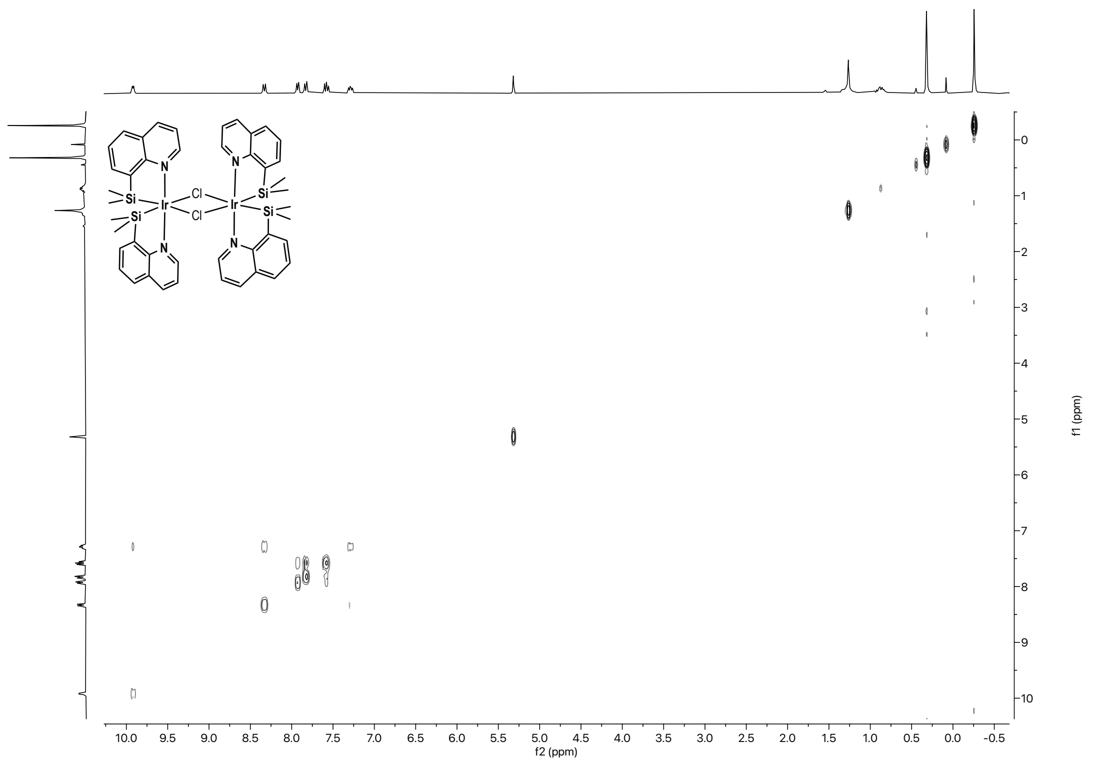

Figure S.3. COSY ^{1}H-^{1}H NMR of **3.**

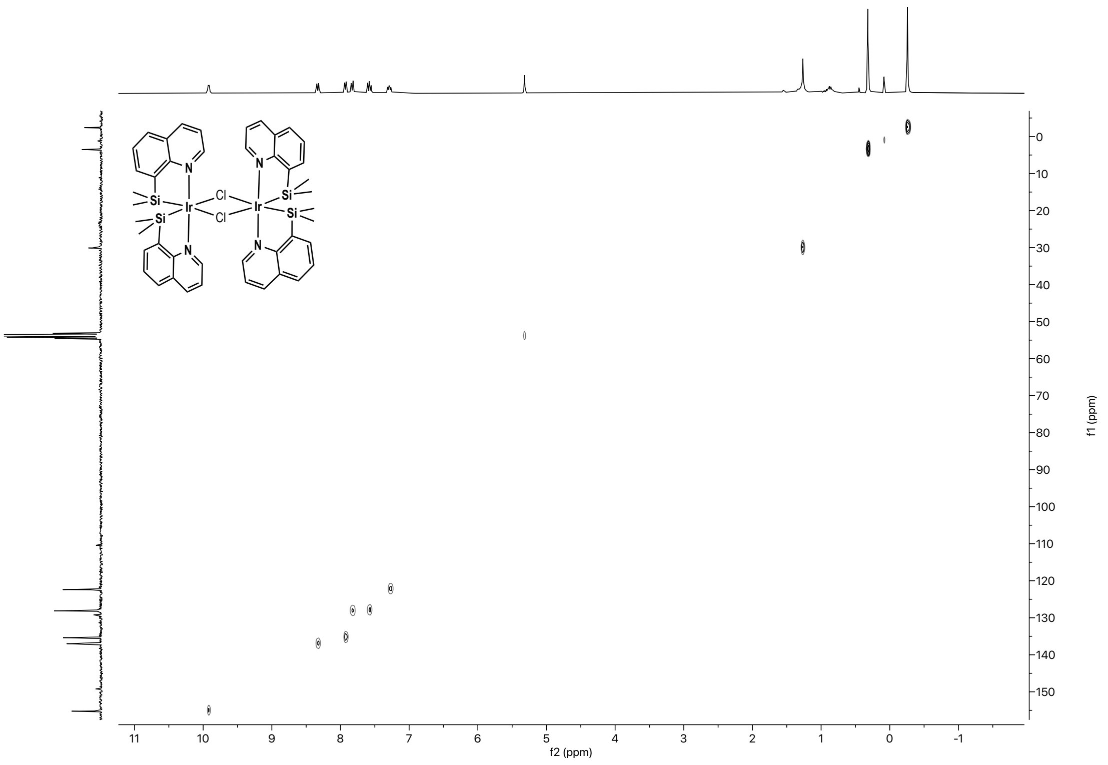

Figure S.4. HSQC ^{1}H-^{13}C NMR of **3.**

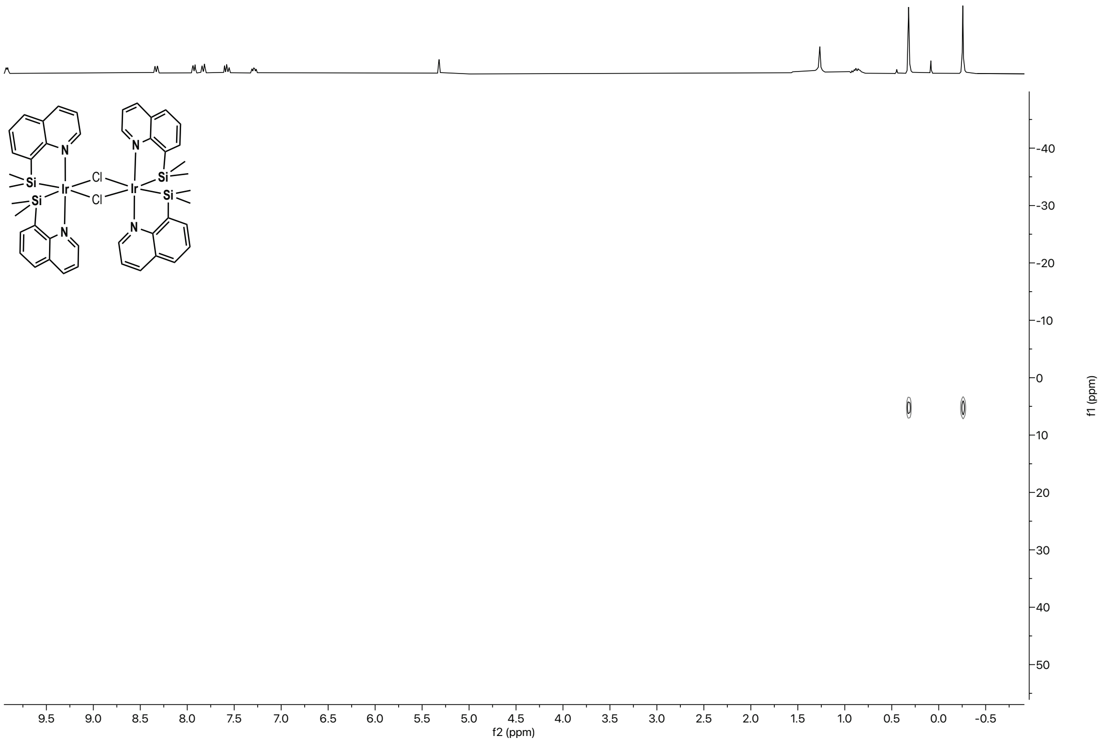

Figure S.5. HMBC ^{1}H-^{29}Si NMR of **3.**

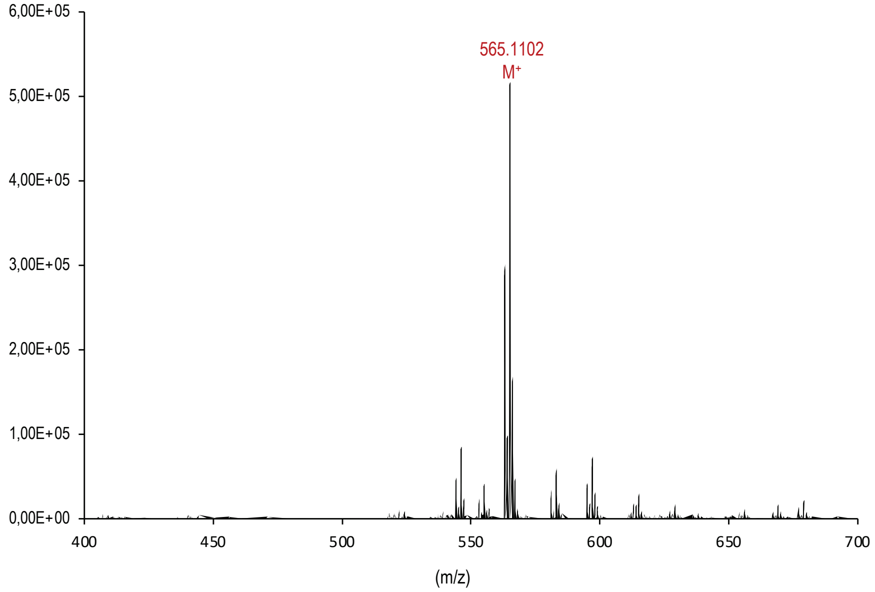

Figure S.6. ESI-MS of **3.**

Synthesis and characterization of 4

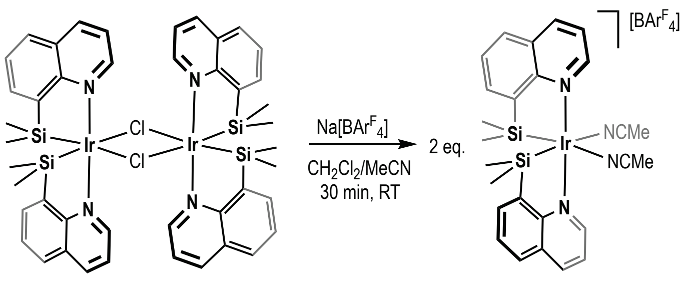

Scheme S.3. Synthesis of **4**.

To a Schlenk charged with **3** (50 mg, 0.042 mmol) and $NaBAr^F_4$ (88 mg, 0.10 mmol), CH_2Cl_2 (5 ml) and MeCN (0.5 mL) were added. The mixture was stirred for 20 minutes and filtrated via cannula. After vacuum concentration, the addition of 20 mL of pentane gave a pale orange solid that was washed with pentane and dried under vacuum.

Characterization of 4

Yield: 101 mg (79%).

^{1}H RMN (500 MHz, CD_2Cl_2): δ 9.31 (s_{broad}, $2H_{arom.}$), 8.44 (d, $J_{3(H-H)}$ = 8.7 Hz, $2H_{arom.}$), 7.99 (d, $J_{3(H-H)}$ = 5.6 Hz, $2H_{arom.}$), 7.91 (d, $J_{3(H-H)}$ = 8.7 Hz, $2H_{arom.}$), 7.73 (s, $8H_{arom.}$, BAr^F_4), 7.67 (m, $2H_{arom.}$), 7.56(s, $4H_{arom.}$, BAr^F_4) 7.45 (s_{broad}, $2H_{arom.}$), 2.21 (s, 6H, MeCN), 0.32 (s, 6H, Si-CH_3), -0.21 (s, 6H, Si-CH_3).

$^{13}C\{^1H\}$ RMN (125 MHz, CD_2Cl_2): δ 161.8 (q, J_{B-C} = 50 Hz, BAr^F_4), 157.1 ($2C_{arom.}$), 153.6 ($2C_{arom.}$), 146.9 ($2C_{arom.}$), 139.2 ($2C_{arom.}$), 136.6 ($2C_{arom.}$), 134.9 (s, BAr^F_4), 132.7 ($2C_{arom.}$), 129.0 (q, J_{F-C} = 12 Hz, BAr^F_4), 128.9 ($2C_{arom.}$), 128.8 ($2C_{arom.}$), 124.7 (q, J_{F-C} = 273 Hz, CF_3), 122.4 ($2C_{arom.}$), 121.0 (2C, NC-CH_3), 117.6 (m, BAr^F_4), 3.1 (2C, MeCN), 2.9 (2C, Si-CH_3), -2.4 (2C, Si-CH_3).

^{29}Si NMR (Chemical shift from ^{1}H-^{29}Si HMBC) (500 MHz, CD_2Cl_2): δ 11.5 ppm.

ESI-MS (MeCN): calc: 565.11; found m/z 565.11. For the ion $[C_{22}H_{24}N_2IrSi_2]^+$.

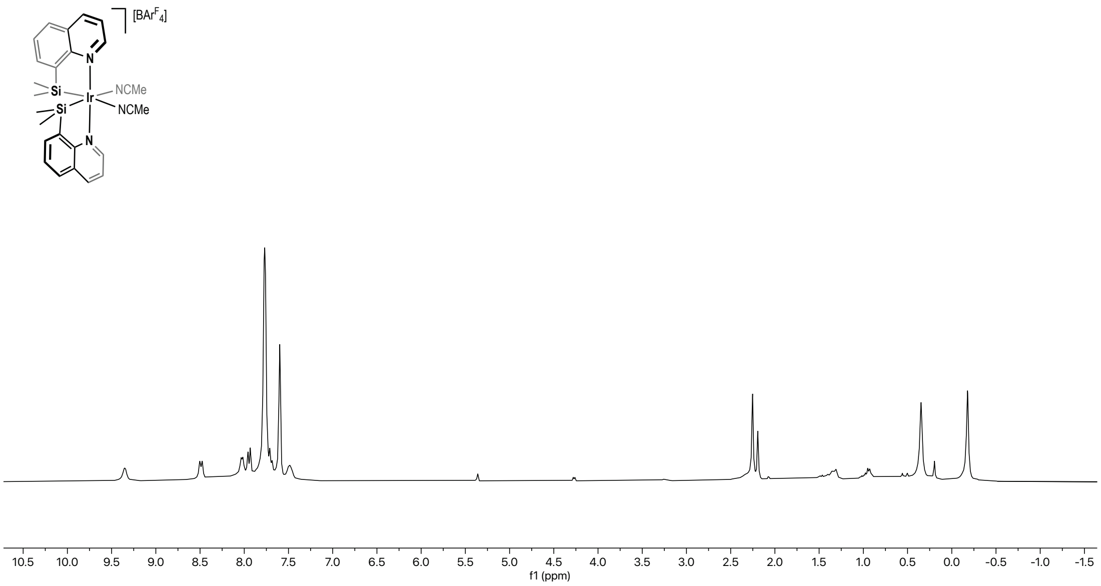

Figure S.7. ^{1}H NMR of **4.**

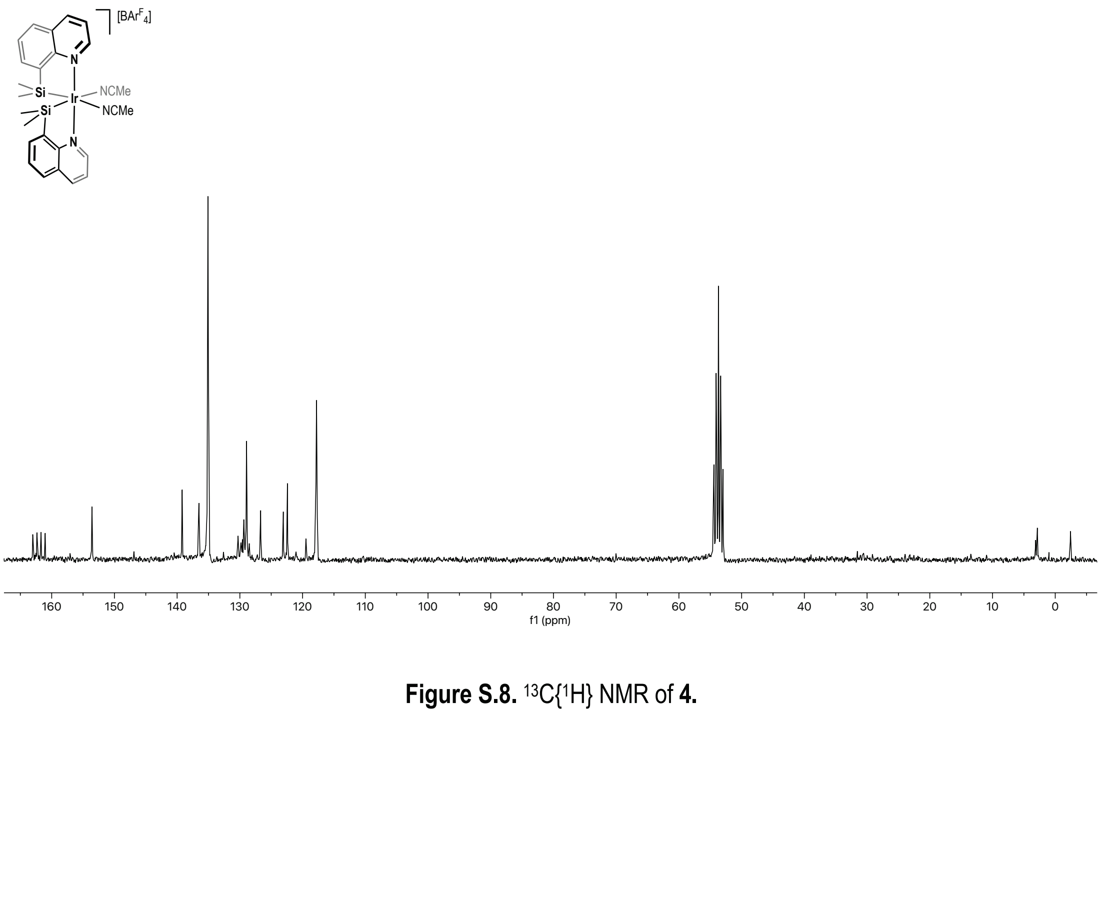

Figure S.8. $^{13}C\{^{1}H\}$ NMR of **4.**

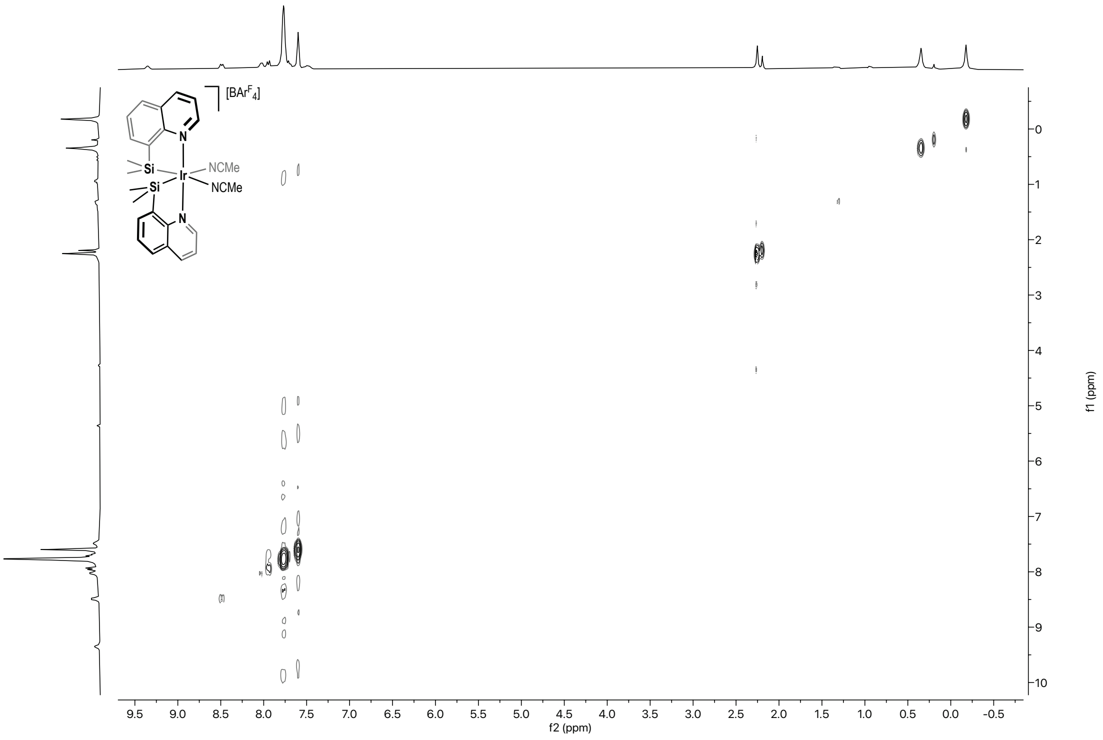

Figure S.9. COSY ^{1}H-^{1}H NMR of **4.**

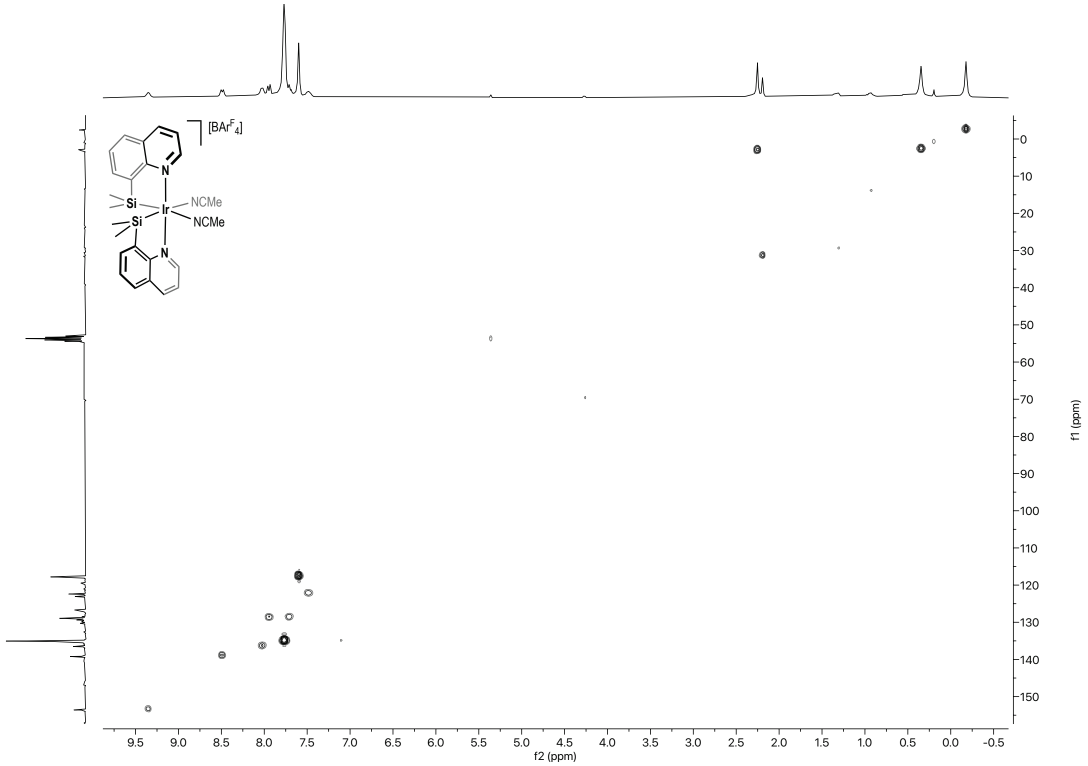

Figure S.10. HSQC ^{1}H-^{13}C NMR of **4.**

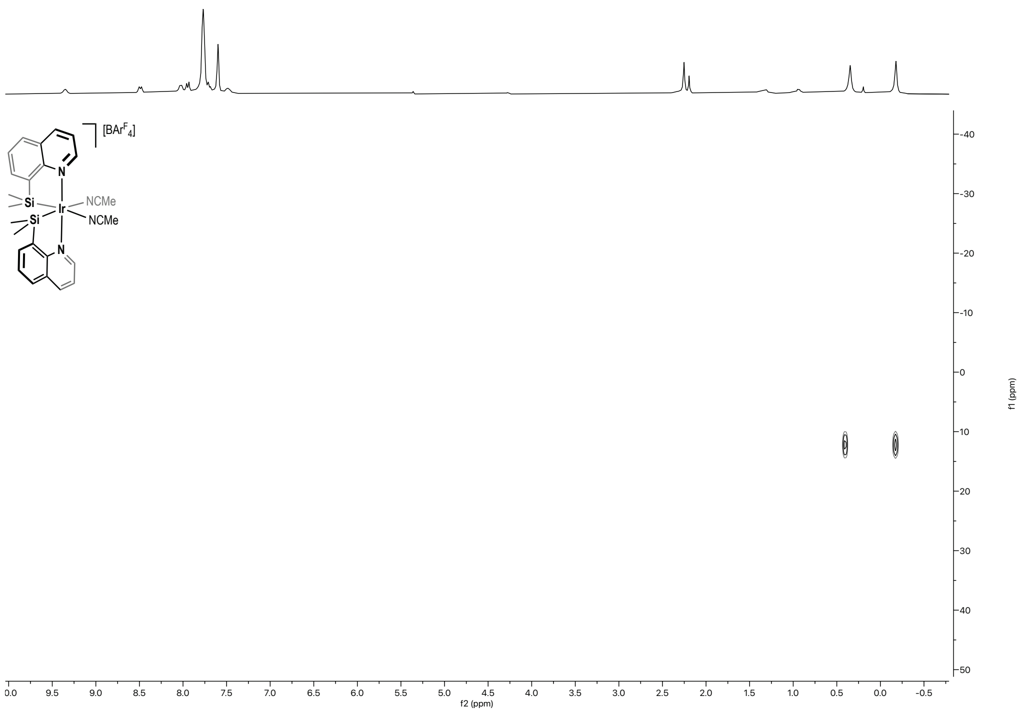

Figure S.11. HMBC ^{1}H-^{29}Si NMR of **4.**

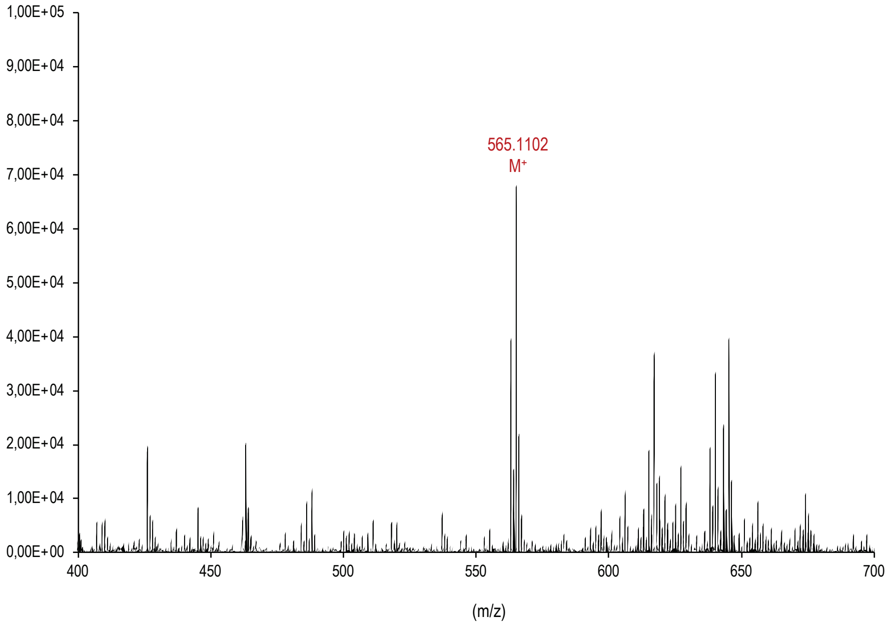

Figure S.12. ESI-MS of **4.**

3. Crystallography

Suitable single crystals of 3 were mounted on a MyTeGen® polymer loo and covered on FOMBLIG oil. Data collection was performed at 150 K on a Bruker D8 VENTURE equipped with MoKα radiation (0.71073 Å) and a Photon3 detector. Temperature was controlled by means of dry nitrogen steam with an Oxford Cryosystem. APEX3 software was employed for data reduction and the absorption correction was performed using SADABS. The crystalline structure was solved and refined by employing SHELXT[6] and SHELXL[7] programs integrated in Olex2 graphical interface.[8] All non-H atoms were refined anisotropically. Hydrogen atoms were placed on calculated positions and refined with idealized geometries. SQUEEZE mask was applied, finding one dichloromethane molecule per unit formula of compound in agreement with the residual electron density. Crystallographic data has been deposited with the Cambridge Crystallographic Data Centre as supplementary publication with no. CCDC 2530286. These data can be obtained free of charge via www.ccdc.cam.ac.uk/data_request/cif, or by emailing data_request@ccdc.cam.ac.uk, or by contacting The Cambridge Crystallographic Data Centre, 12 Union Road, Cambridge, CB2 1EZ, UK; fax: +44 1223 336033. e-mail: deposit@ccdc.cam.ac.uk or http://www.ccdc.cam.ac.uk).

Table S1. Crystallographic data and structure refinement details of all compounds.

Identification code	**3**
CCDC	2530286
Empirical formula	$C_{45}H_{50}Cl_4Ir_2N_4Si_4$
Formula weight	1285.45
Temperature/K	150.00
Crystal system	monoclinic
Space group	C2/c
a/Å	12.4258(4)
b/Å	15.5584(5)
c/Å	24.9298(9)
α/°	90
β/°	91.0330(10)
γ/°	90
Volume/Å^3	4818(3)
Z	4
ρ_{calc}g/cm^3	1.772
μ/mm^{-1}	5.875
F(000)	2504.0
Crystal size/mm^3	0.08 × 0.075 × 0.005
Radiation	MoKα (λ = 0.71073)
2Θ range for data collection/°	4.196 to 52.766
Index ranges	-15 ≤ h ≤ 15, -19 ≤ k ≤ 19, -31 ≤ l ≤ 31
Reflections collected	40295
Independent reflections	4949 [R_{int} = 0.0264, R_{sigma} = 0.0155]
Data/restraints/parameters	4949/0/257
Goodness-of-fit on F^2	1.183
Final R indexes [I>=2σ (I)]	R_1 = 0.0223, wR_2 = 0.0557
Final R indexes [all data]	R_1 = 0.0242, wR_2 = 0.0568
Largest diff. peak/hole / e Å^{-3}	1.44/-1.05

Table S2. Selected bond lengths of compound **3**.

Atom	Atom	Length/Å	Atom	Atom	Length/Å
Ir1	Cl1[1]	2.6473(9)	Ir1	Cl1	2.5792(9)
Ir1	Si1	2.2814(1)	Ir1	Si2	2.2882(1)
Ir1	N1	2.092(3)	Ir1	N2	2.041(3)

[1]1-X,+Y,1/2-Z

Table S3. Selected bond angles of compound **3**.

Atom	Atom	Atom	Angle/˚	Atom	Atom	Atom	Angle/˚
Cl1	Ir1	Cl1[1]	77.99(3)	N1	Ir1	Si2	90.63(10)
Si2	Ir1	Cl1	169.92(4)	N1	Ir1	Si1	85.17(10)
Si2	Ir1	Cl1[1]	92.98(3)	N2	Ir1	Cl1[1]	95.01(10)
Si1	Ir1	Cl1	98.20(3)	N2	Ir1	Cl1	90.64(10)
Si1	Ir1	Cl1[1]	174.97(4)	N2	Ir1	Si2	85.64(10)
Si1	Ir1	Si2	91.06(4)	N2	Ir1	Si1	88.28(10)
N1	Ir1	Cl1[1]	91.79(9)	N2	Ir1	N1	172.40(13)
N1	Ir1	Cl1	94.09(9)				

[1]1-X,+Y,1/2-Z

4. Catalytic experiments

4.1. Catalytic carbene insertion from ethyl diazoacetate into triethylsilane (Table 1 in manuscript).

Reaction Conditions: Ethyl diazoacetate (0.1 mmol), triethylsilane (0.5 mmol), 5 mol % of catalyst in 2 mL of dichloromethane at 25 °C. Catalyst and triethylsilane were dissolved in 1 mL of CH_2Cl_2, then ethyl diazoacetate solved in 1 mL of CH_2Cl_2 was added slowly (during 6 hours) over the triethylsilane/catalyst solution under argon. After complete addition, the final mixture was left to react for another 6 hours. After 12 hours of reaction, the solvents were removed under reduced pressure, then 0.1 mmol of p-iodoanisole (internal standard) was added and a ^1H NMR experiment was performed to calculate the conversion.
The α-silylether obtained (ethyl 2-(triethylsilyl)acetate) was identified by ^1H NMR by comparison of the spectra with those found in the literature.[9]

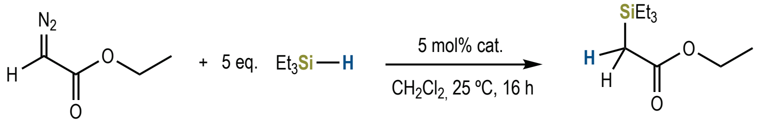

Scheme S.4. Catalytic reaction for the synthesis of ethyl 2-(triethylsilyl)acetate

Catalyst: **2**

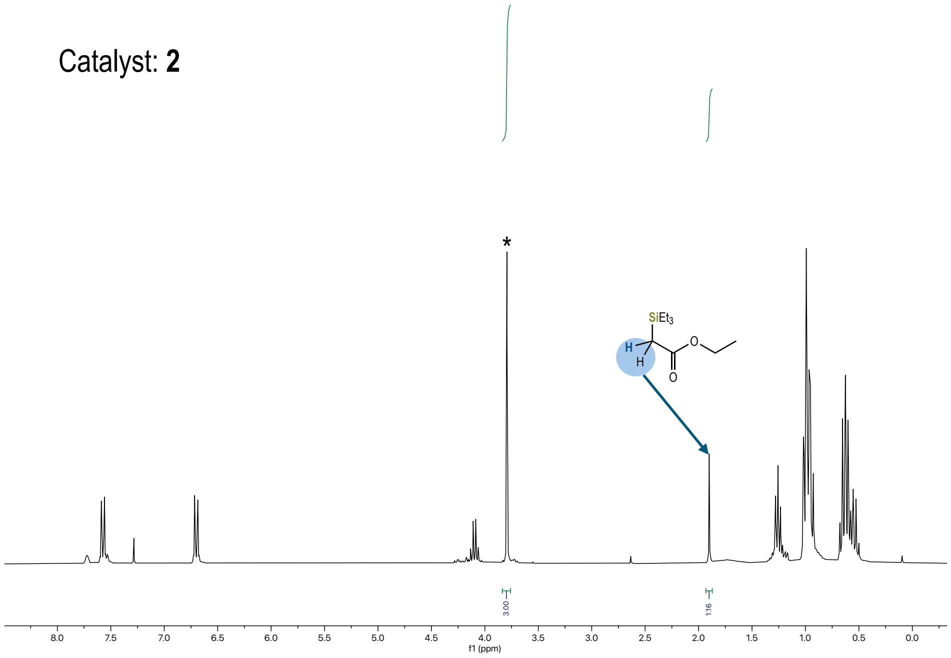

Figure S.13. ^1H NMR of the reaction crude of the carbene insertion from ethyl diazoacetate into triethylsilane catalyzed by **2**. (*) p-iodoanisole (internal standard).

Catalyst: **4**

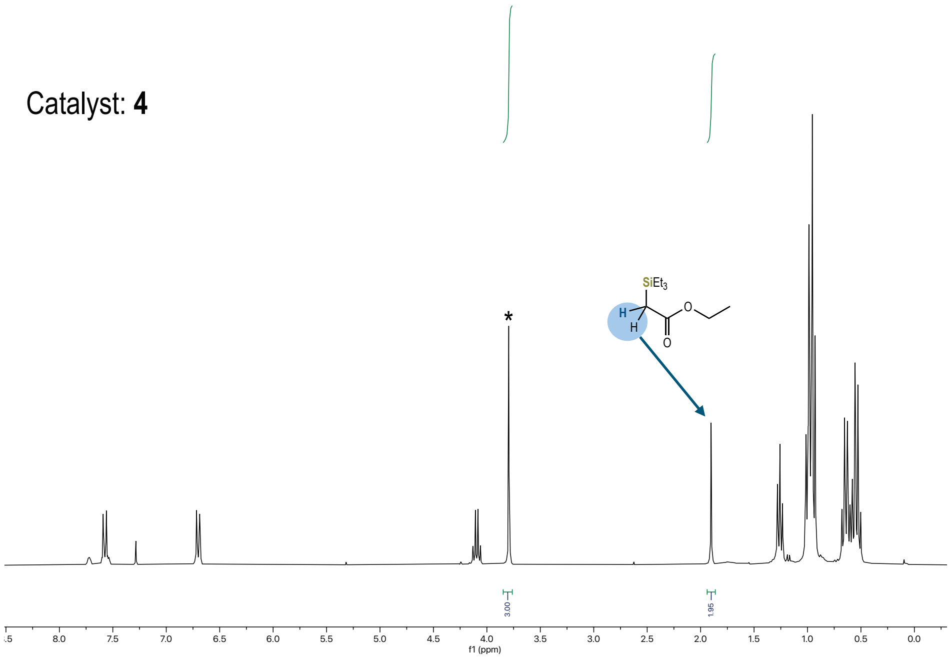

Figure S.14. ^{1}H NMR of the reaction crude of the carbene insertion from ethyl diazoacetate into triethylsilane catalyzed by **4**. (*) p-iodoanisole (internal standard).

4.2. Catalytic carbene insertion from ethyl diazoacetate into other silanes (Table 2 in manuscript).

Reaction Conditions: Ethyl diazoacetate (0.1 mmol), hydrosilanes (0.5 mmol), 5 mol % of catalyst in 2 mL of dichloromethane at 25 °C. Catalyst and silane were dissolved in 1 mL of CH_2Cl_2, then ethyl diazoacetate solved in 1 mL of CH_2Cl_2 was added slowly (during 6 hours) over the triethylsilane/catalyst solution under argon. After complete addition, the final mixture was left to react for another 6 hours. After 12 hours of reaction, the solvents were removed under reduced pressure, then 0.1 mmol of p-iodoanisole (internal standard) was added and a ^{1}H NMR experiment was performed to calculate the conversion.

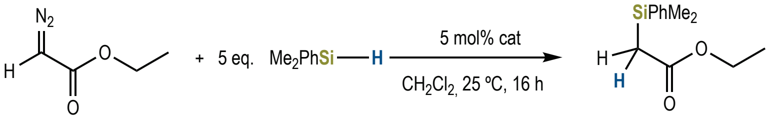

Scheme S.5. Catalytic reaction for the synthesis of ethyl 2-(dimethylphenylsilyl)acetate

Catalyst: **2**

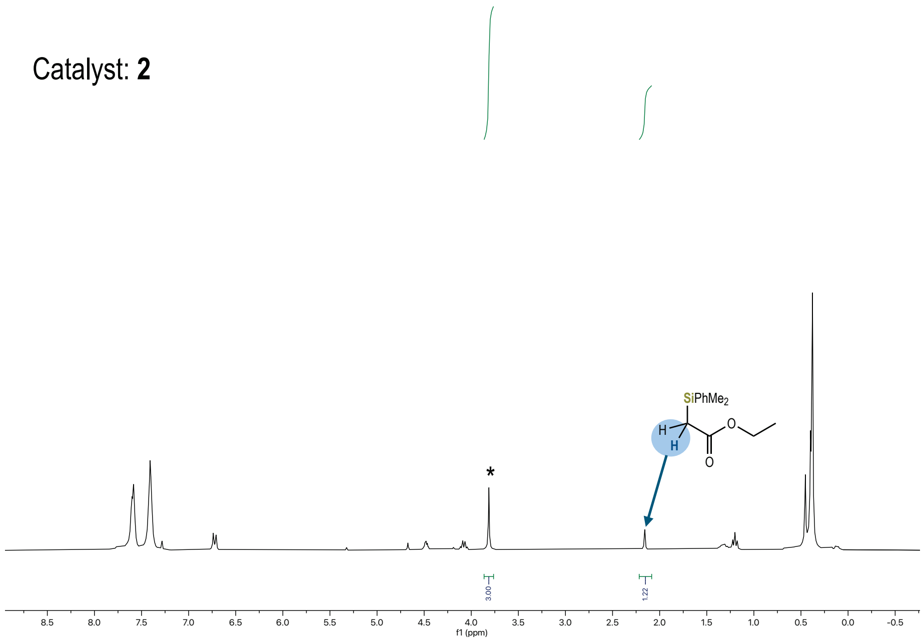

Figure S.15. ^{1}H NMR of the reaction crude of the carbene insertion from ethyl diazoacetate into dimethylphenylsilane catalyzed by **2**. (*) p-iodoanisole (internal standard).

Catalyst: **4**

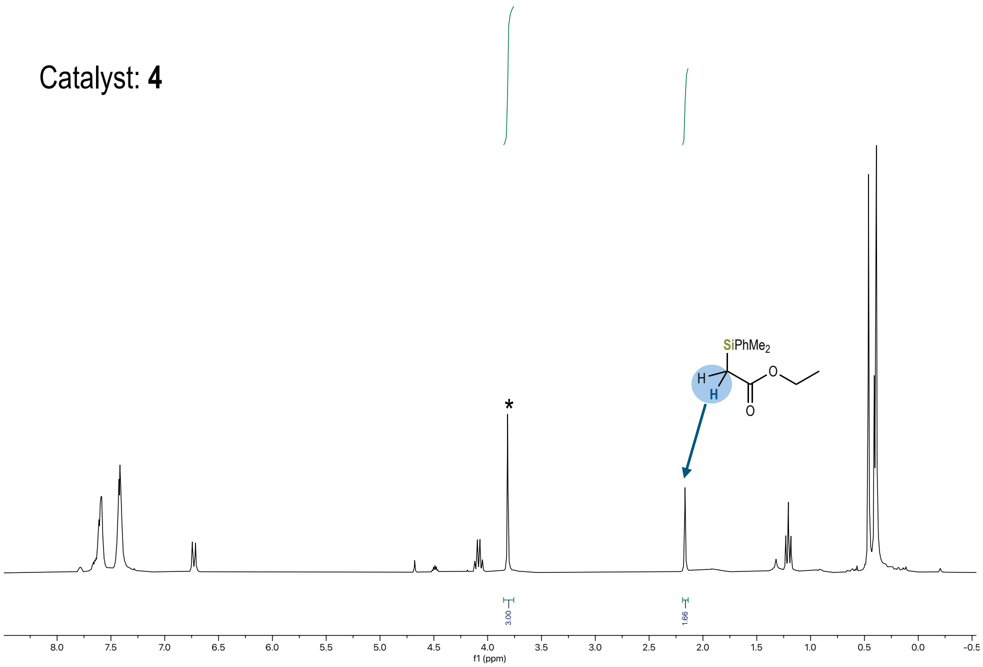

Figure S.16. ^{1}H NMR of the reaction crude of the carbene insertion from ethyl diazoacetate into dimethylphenylsilane catalyzed by **4**. (*) p-iodoanisole (internal standard).

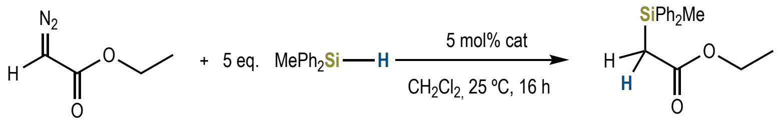

Scheme S.6. Catalytic reaction for the synthesis of ethyl 2-(diphenylmethylsilyl)acetate

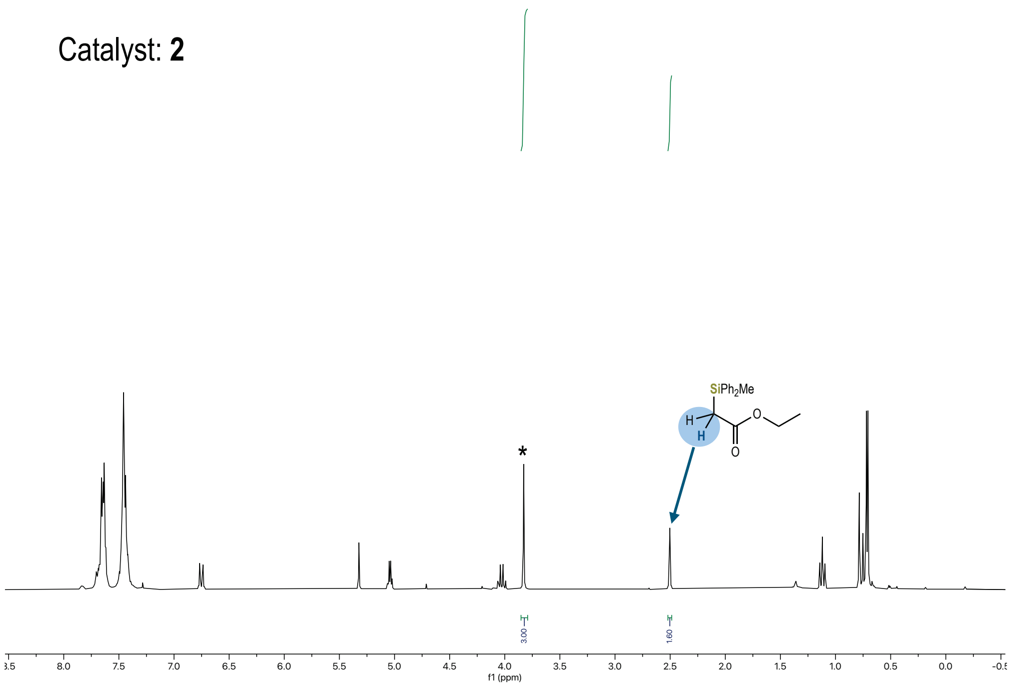

Figure S.17. ^{1}H NMR of the reaction crude of the carbene insertion from ethyl diazoacetate into methyldiphenylsilane catalyzed by **2**. (*) p-iodoanisole (internal standard).

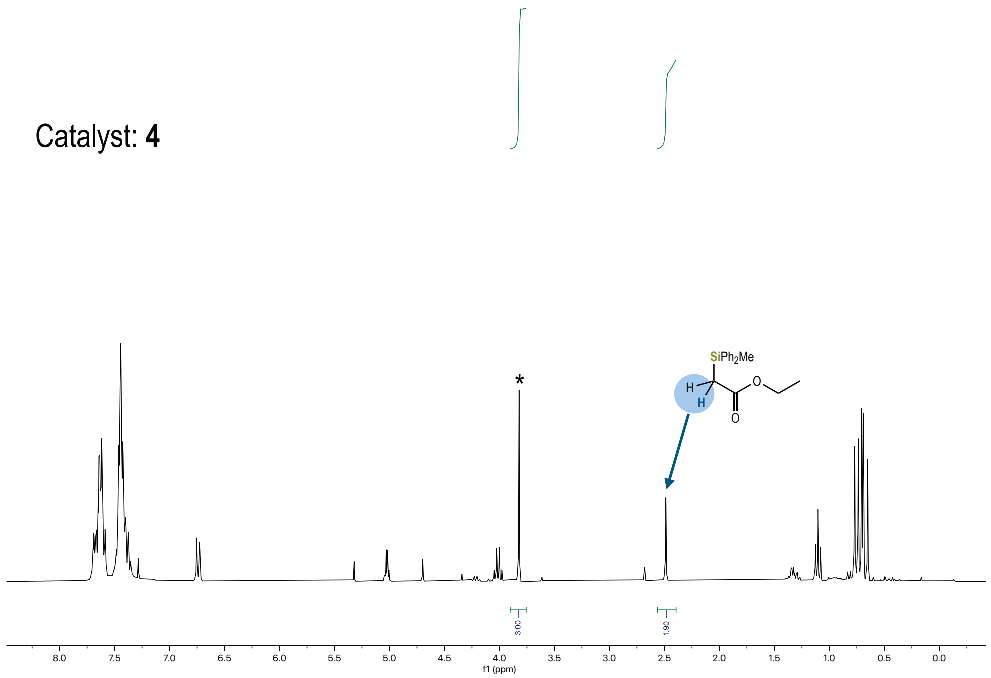

Figure S.18. ^{1}H NMR of the reaction crude of the carbene insertion from ethyl diazoacetate into methyldiphenylsilane catalyzed by **4**. (*) p-iodoanisole (internal standard).

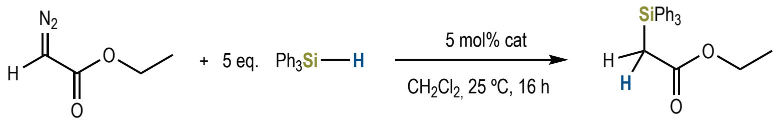

Scheme S.7. Catalytic reaction for the synthesis of ethyl 2-(triphenylsilyl)acetate

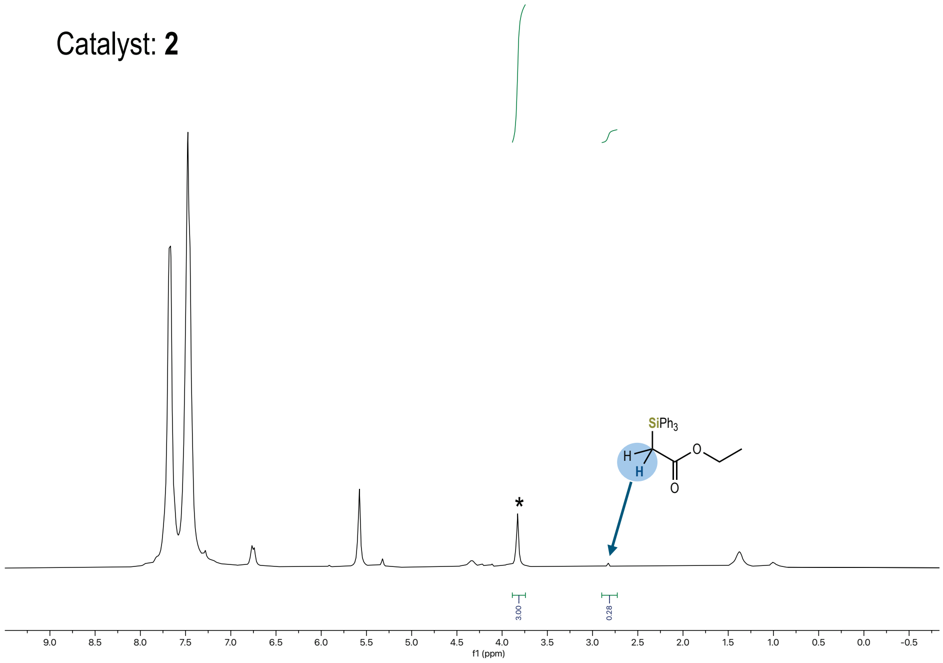

Figure S.19. ^{1}H NMR of the reaction crude of the carbene insertion from ethyl diazoacetate into triphenylsilane catalyzed by **2**. (*) p-iodoanisole (internal standard).

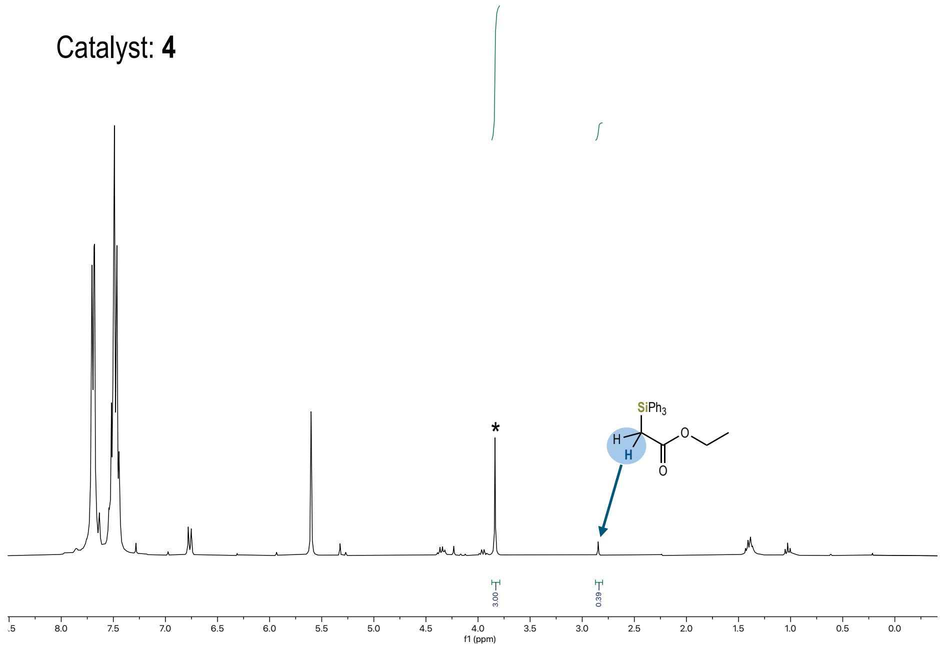

Figure S.20. ^{1}H NMR of the reaction crude of the carbene insertion from ethyl diazoacetate into triphenylsilane catalyzed by **4**. (*) p-iodoanisole (internal standard).

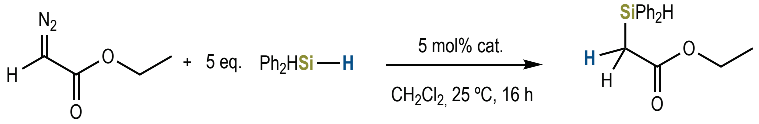

Scheme S.8. Catalytic reaction for the synthesis of ethyl 2-(diphenylsilyl)acetate

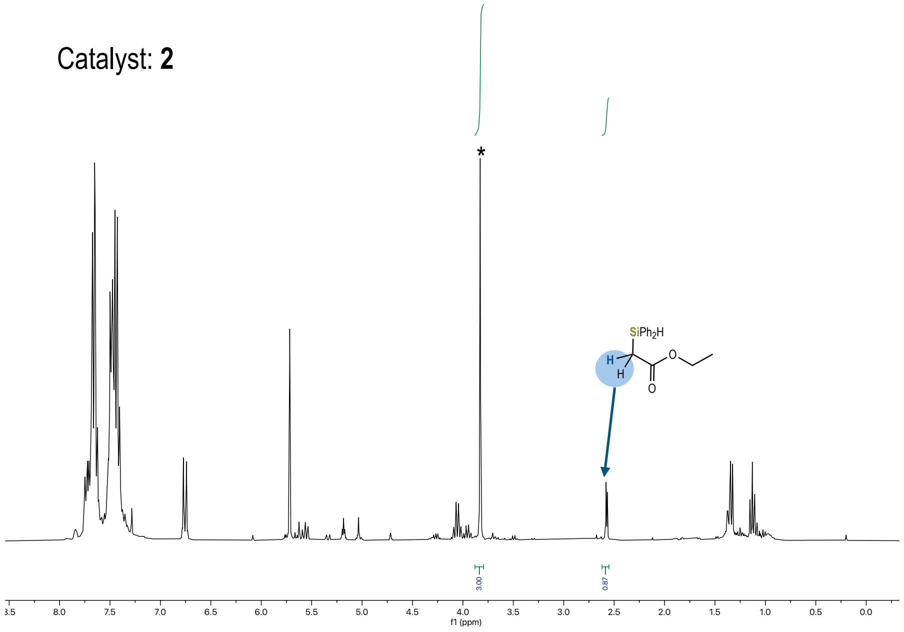

Figure S.21. ^{1}H NMR of the reaction crude of the carbene insertion from ethyl diazoacetate into diphenylsilane catalyzed by **2**. (*) p-iodoanisole (internal standard).

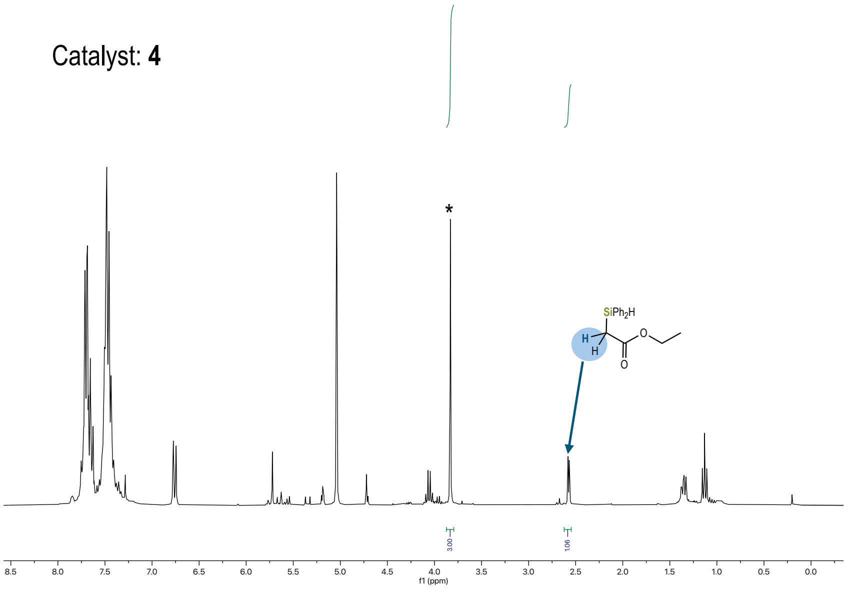

Figure S.22. ^{1}H NMR of the reaction crude of the carbene insertion from ethyl diazoacetate into diphenylsilane catalyzed by **4**. (*) p-iodoanisole (internal standard).

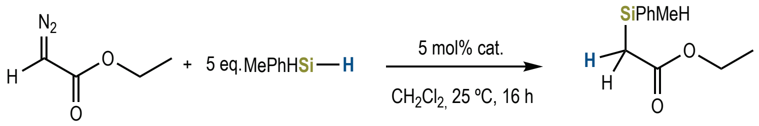

Scheme S.9. Catalytic reaction for the synthesis of ethyl 2-(methylphenylsilyl)acetate

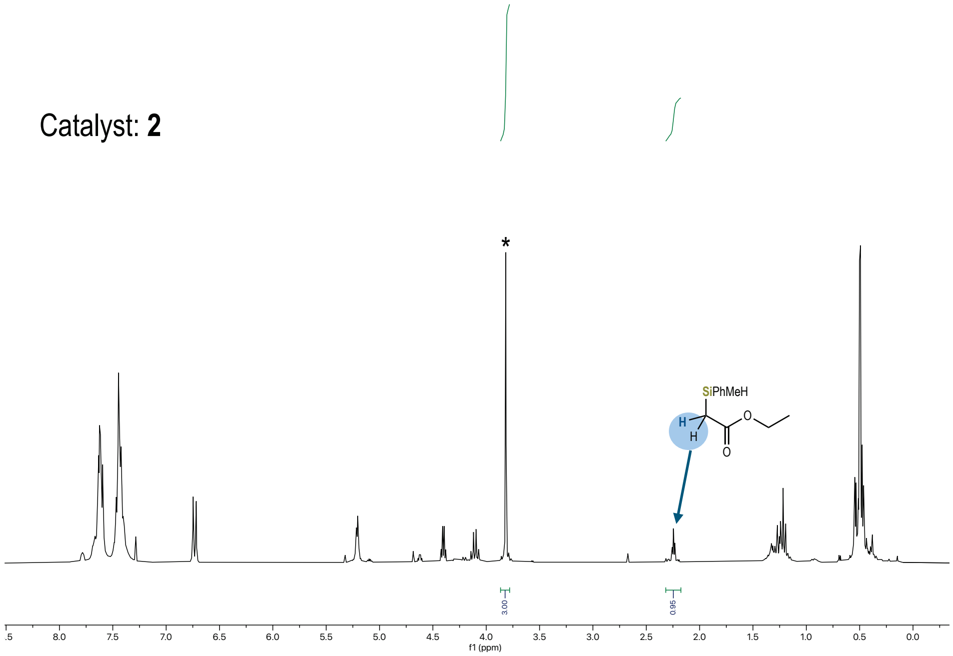

Figure S.23. ^{1}H NMR of the reaction crude of the carbene insertion from ethyl diazoacetate into methylphenylsilane catalyzed by **2**. (*) p-iodoanisole (internal standard).

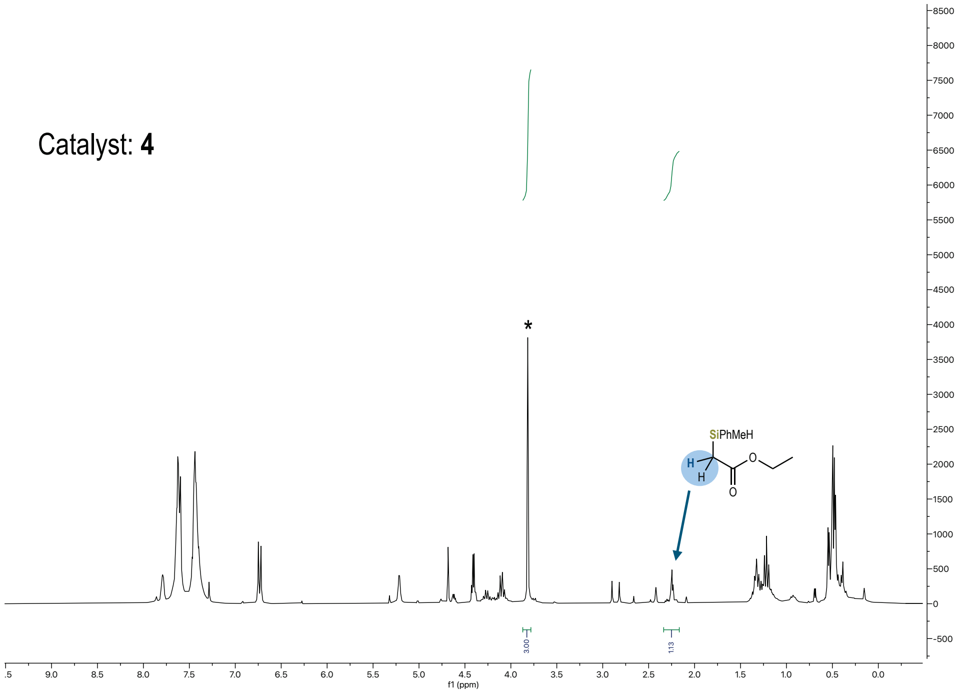

Figure S.24. ^{1}H NMR of the reaction crude of the carbene insertion from ethyl diazoacetate into methylphenylsilane catalyzed by **4**. (*) p-iodoanisole (internal standard).

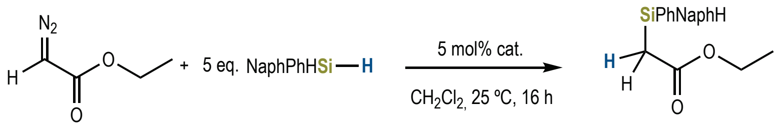

Scheme S.10. Catalytic reaction for the synthesis of ethyl 2-(naphthylphenylsilyl)acetate

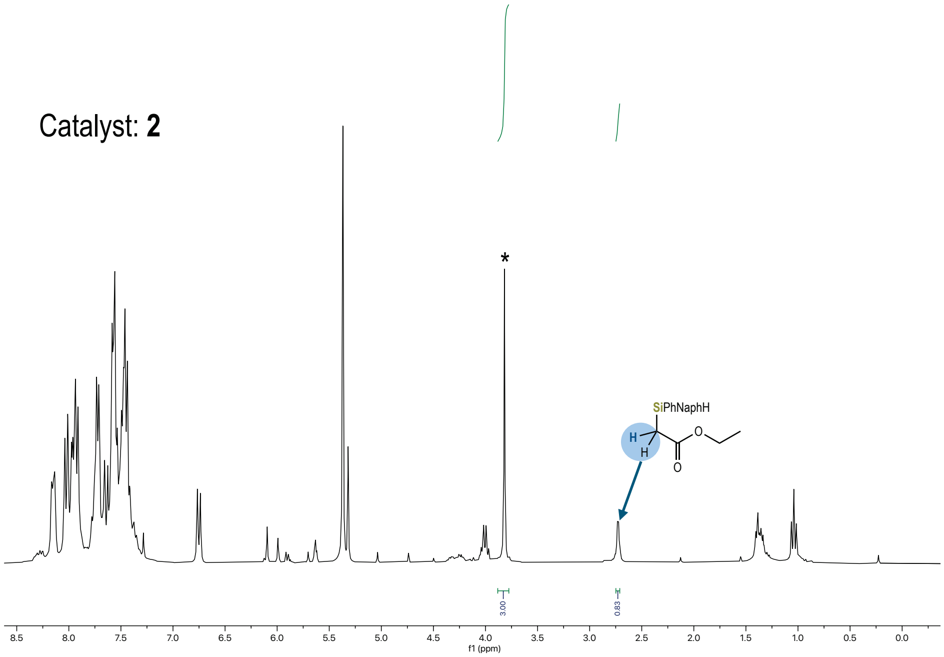

Figure S.25. ^{1}H NMR of the reaction crude of the carbene insertion from ethyl diazoacetate into naphthylphenylsilane catalyzed by **2**. (*) p-iodoanisole (internal standard).

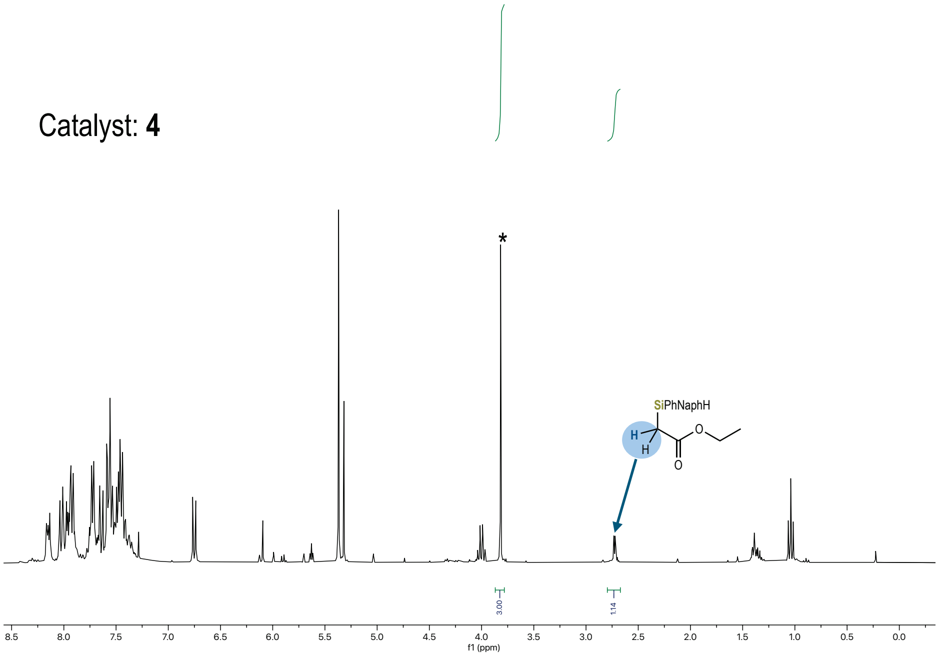

Figure S.26. ^{1}H NMR of the reaction crude of the carbene insertion from ethyl diazoacetate into naphthylphenylsilane catalyzed by **4**. (*) p-iodoanisole (internal standard).

4.3. Catalytic carbene insertion from other diazo esters into triethylsilane (Table 2 in manuscript).

Reaction Conditions: diazo ester (methyl 2-diazopropanoate and methyl 2-diazophenylacetate 0.1 mmol), triethylsilane (0.5 mmol), 5 mol % of catalyst in 2 mL of dichloromethane at 25 °C. Catalyst and silane were dissolved in 1 mL of CH_2Cl_2, then ethyl diazoacetate solved in 1 mL of CH_2Cl_2 was added slowly (during 6 hours) over the triethylsilane/catalyst solution under argon. After complete addition, the final mixture was left to react for another 6 hours. After 12 hours of reaction, the solvents were removed under reduced pressure, then 0.1 mmol of p-iodoanisole (internal standard) was added and a ^{1}H NMR experiment was performed to calculate the conversion.

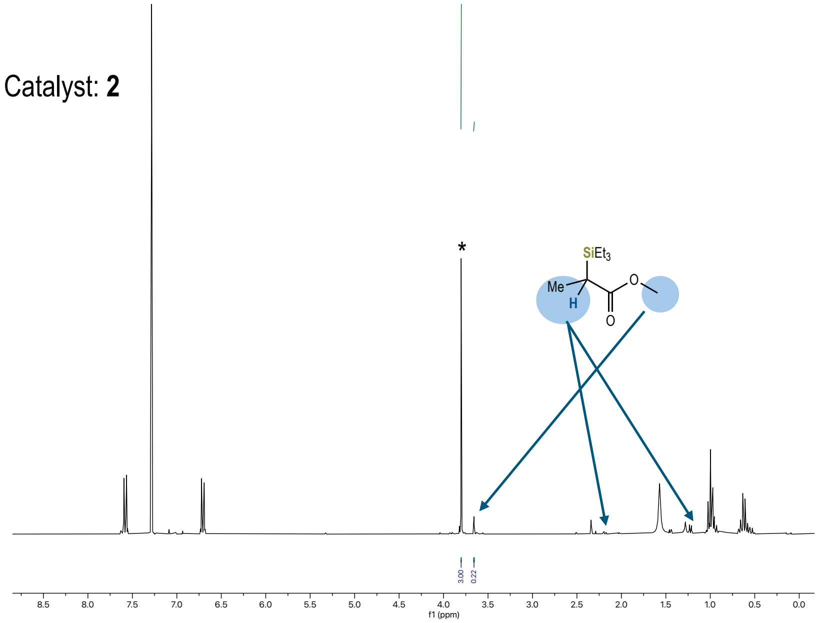

Figure S.27. ^{1}H NMR of the reaction crude of the carbene insertion from methyl 2-diazopropanoate into triethylsilane catalyzed by **2**. (*) p-iodoanisole (internal standard).

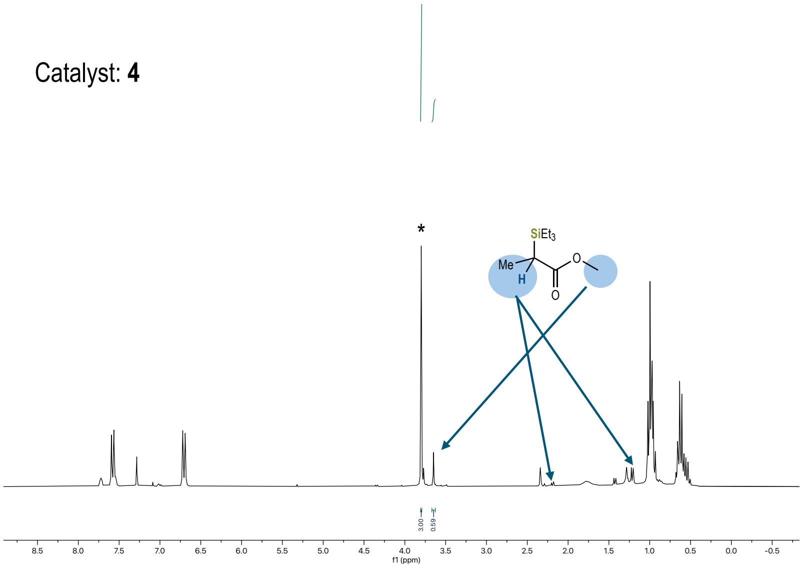

Figure S.28. ^{1}H NMR of the reaction crude of the carbene insertion from methyl 2-diazopropanoate into triethylsilane catalyzed by **4**. (*) p-iodoanisole (internal standard).

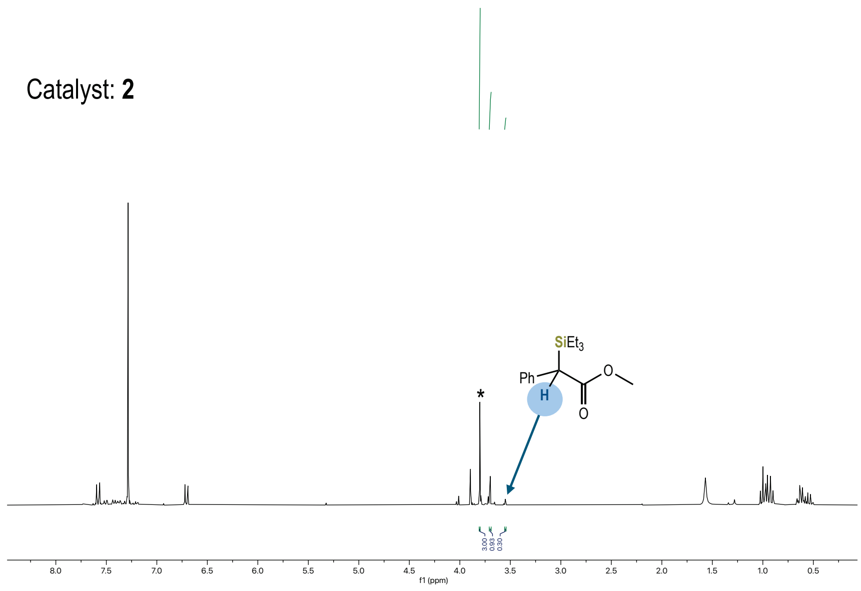

Figure S.29. ^{1}H NMR of the reaction crude of the carbene insertion from methyl 2-diazophenylacetate into triethylsilane catalyzed by **2**. (*) p-iodoanisole (internal standard).

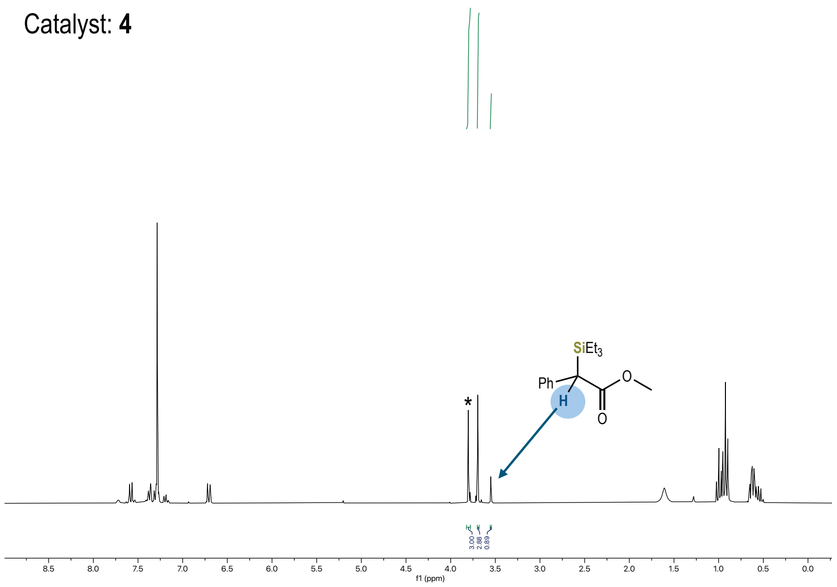

Figure S.30. ^{1}H NMR of the reaction crude of the carbene insertion from methyl 2-diazophenylacetate into triethylsilane catalyzed by **4**. (*) p-iodoanisole (internal standard).

5. Competition experiment for the investigation of the kinetic isotope effect (KIE)

Ethyl diazoacetate (0.10 mmol), triethylsilane (0.25 mmol), triethylsilane-d (0.25 mmol), and catalyst **2** (5 mol%) in dichloromethane (2 mL) at 25 °C. Catalyst **2** and the silane reagents were dissolved in dichloromethane (1 mL), and a solution of ethyl diazoacetate in dichloromethane (1 mL) was added dropwise over 6 h. Upon completion of the addition, the reaction mixture was analyzed by ^{1}H NMR spectroscopy in $CDCl_3$.

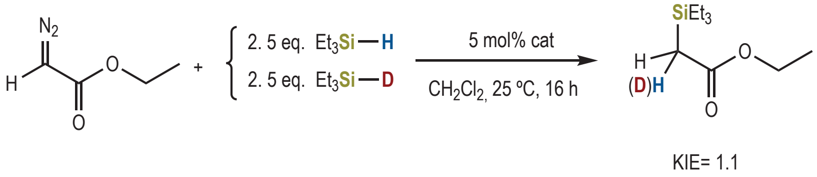

Scheme S.11. Catalytic reaction for KIE determination.

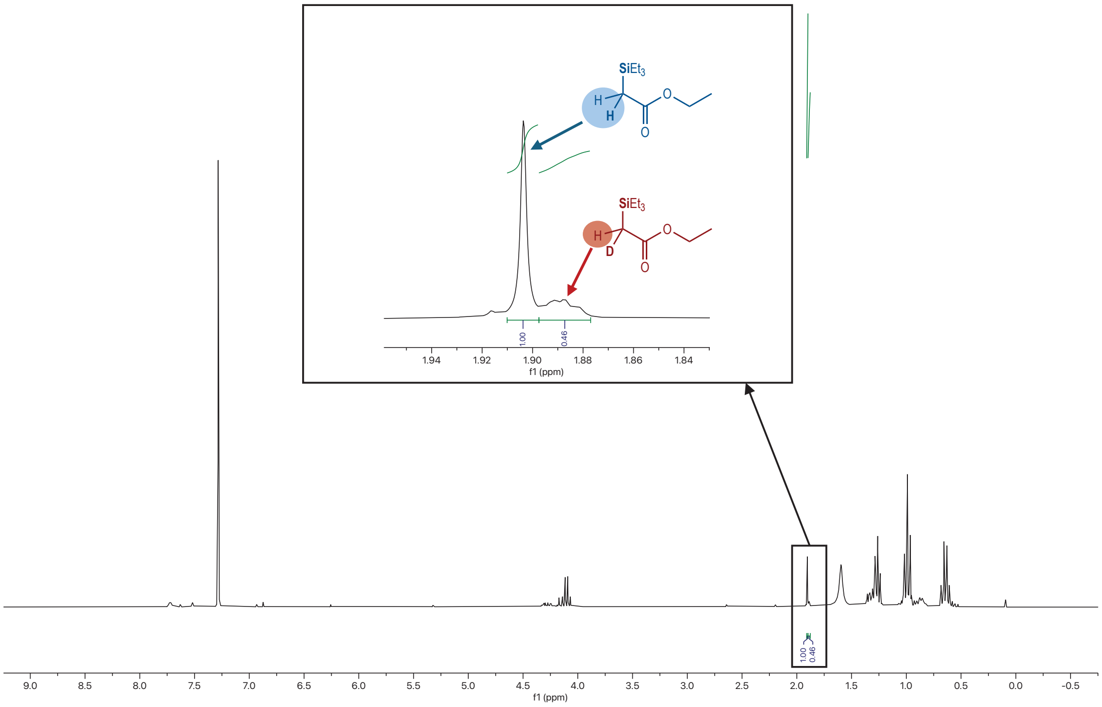

Figure S.31. ^{1}H NMR of the reaction crude of the carbene insertion from ethyl diazoacetate into Et_3Si–H and Et_3Si–D catalyzed by **2**.

6. 1 mmol-scale experiment

Ethyl diazoacetate (1.00 mmol), triethylsilane (5 mmol), and catalyst **2** (5 mol%) in dichloromethane (2 mL) at 25 °C. Catalyst and silane were dissolved in 2 mL of CH_2Cl_2, then ethyl diazoacetate solved in 2 mL of CH_2Cl_2 was added slowly (during 6 hours) over the triethylsilane/catalyst solution under argon. After complete addition, the final mixture was left to react for another 6 hours. After 12 hours of reaction, the solvents were removed under reduced pressure and a ^{1}H NMR experiment was performed. The product, ethyl 2-(triethylsilyl)acetate, was purified by column chromatography using SiO_2 as the stationary phase and hexane/ethyl acetate (9:1) as eluents. A yellow oil (99 mg, 49% yield) was obtained.

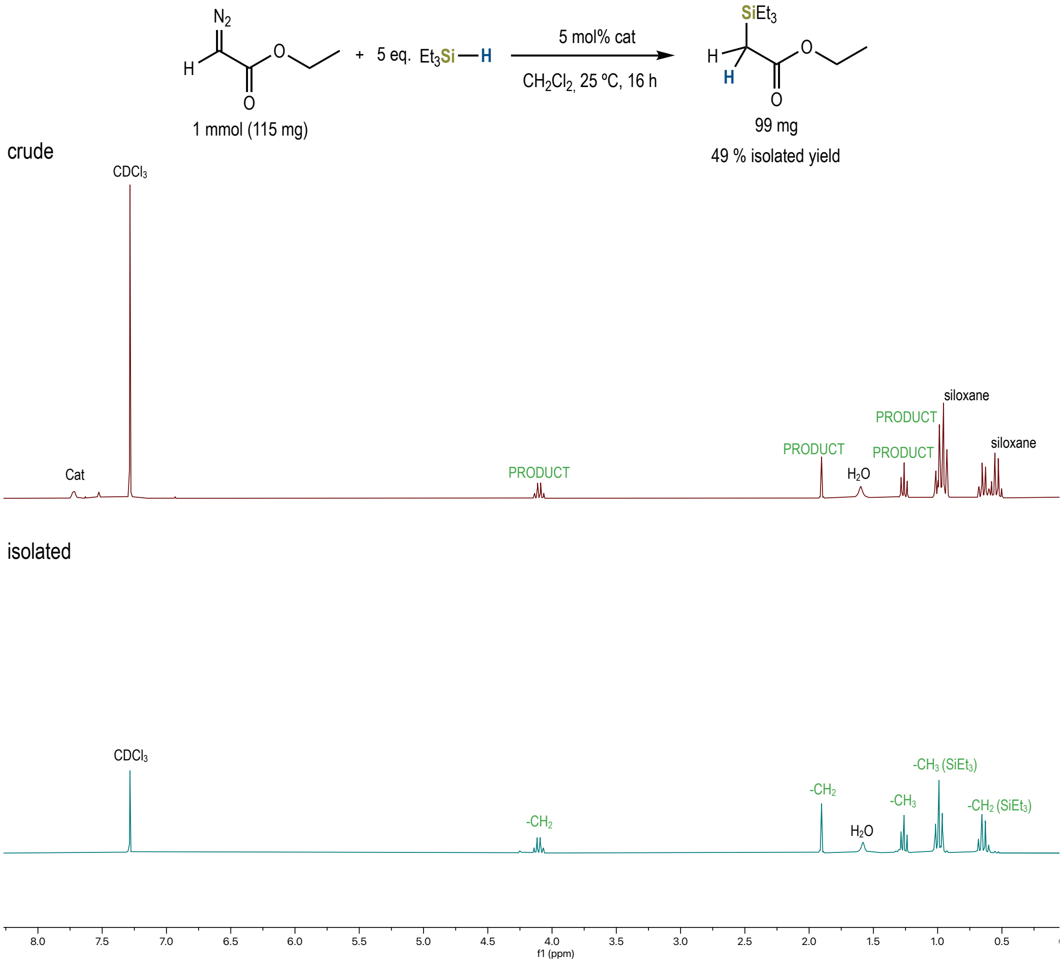

Figure S.32. ^{1}H NMR of the reaction crude of the carbene insertion from ethyl diazoacetate into Et_3Si–H catalyzed by **2** (up). ^{1}H NMR of the isolated product (bottom).

6. References

(1) Bennet, M. A.; Saxby, J. D. Cyclooctatetraene-Rhodium(I) Complexes. *Inorg. Chem.* **1967**, *7*, 321-324.

(2) Herde, J. L.; Senoff, C. V. μ-Dichlorotetrakis(cyclooctene) diriidium(I). *Inorg. Nucl. Chem. Lett*. **1971**, *7*, 1029.

(3) Brookhart, M.; Grant, B.; Volpe Jr., A. F. $[(3,5\text{-}(CF_3)_2C_6H_3)_4B]^{-}[H(OEt_2)_2]^{+}$: A convenient reagent for generation and stabilization of cationic, highly electrophilic organometallic complexes. *Organometallics* **1992**, *11*, 3920.

(4) Prieto-Pascual, U.; Martínez de Morentin, A.; Choquesillo-Lazarte, D.; Rodriguez-Dieguez, A.; Freixa, Z.; Huertos, M. A. Catalytic activation of remote alkenes through silyl-rhodium(III) complexes. *Dalton Trans*., **2023***, 52*, 8990.

(5) Prieto-Pascual, U.; Alli, I. V.; Bustos, I.; Vitorica-Yrezabal, I. J.; Matxain, J. M.; Freixa, Z.; Huertos, M. A. Air-stable 14-electron rhodium(III) complexes bearing Si,N ligands as catalysis in hydrolysis of silanes. *Organometallics* **2023**, *42*, 2991-2998.

(6) Sheldrick, G. M. *Acta Cryst. Sect. A Found. Adv.*, **2015**, *71,* 3–8

(7) Sheldrick, G. M. *Acta Cryst. Sect. C Struct. Chem.*, **2015**, *71*, 3–8.

(8) Dolomanov, O. V.; Bourhis, L. J.; Gildea, R. J.; Howard, J. A. K.; Puschmann, H. *J. Appl. Cryst.*, **2009**, *42*, 339–341.

(9) Kidonakis, M.; Stratakis, M. Au nanoparticle-catalyzed insertion of carbenes from α-diazocarbonyl compounds into hydrosilanes. *Org. Lett.* **2018**, *20*, 4086-4089.
